# Histamine N-methyltransferase (HNMT) as a potential auxiliary biomarker for predicting adaptability to anti-HER2 drug treatment in breast cancer patients

**DOI:** 10.1186/s40364-024-00715-5

**Published:** 2025-01-09

**Authors:** Tzu-Chun Cheng, Mien-Chie Hung, Lu-Hai Wang, Shih-Hsin Tu, Chih-Hsiung Wu, Yun Yen, Chi-Long Chen, Jacqueline Whang-Peng, Wen-Jui Lee, You-Cheng Liao, Yu-Ching Lee, Min-Hsiung Pan, Hui-Kuan Lin, Huey-En Tzeng, Peixuan Guo, Cheng-Ying Chu, Li-Ching Chen, Yuan-Soon Ho

**Affiliations:** 1 Institute of Biochemistry and Molecular Biology, College of Life Sciences, China Medical University, Taichung, Taiwan; 2 Graduate Institute of Biomedical Sciences, Institute of Biochemistry and Molecular Biology, Research Center for Cancer Biology, Cancer Biology and Precision Therapeutics Center, and Center for Molecular Medicine, China Medical University, Taichung, Taiwan; 3 Department of Biotechnology, Asia University, Taichung, Taiwan; 4 Institute of Integrated Medicine and Chinese Medicine Research Center, China Medical University, Taichung, Taiwan; 5 Department of Surgery, School of Medicine, College of Medicine, Taipei Medical University; & Department of Surgery, Taipei Medical University Hospital, Taipei, Taiwan; 6 TMU Research Center of Cancer Translational Medicine, Taipei Medical University, Taipei, Taiwan; 7 Department of Pathology, School of Medicine, College of Medicine, Taipei Medical University, Taipei, Taiwan; 8 Department of Pathology, Taipei Medical University Hospital, Taipei Medical University, Taipei, Taiwan; 9 Ph.D. Program in Medical Neuroscience, College of Medical Science and Technology, Taipei Medical University, Taipei, Taiwan; 10 Institute of Food Sciences and Technology, National Taiwan University, Taipei, Taiwan; 11 Department of Pathology, Duke University Medical Center, Duke University School of Medicine, Durham, NC 27710 USA; 12 Division of Hematology/Medical Oncology, Department of Medicine, Taichung Veterans General Hospital, Taichung City, Taiwan; 13 Department of Post-Baccalaureate Medicine, College of Medicine, National Chung-Hsing University, Taichung, Taiwan; 14 Center for RNA Nanobiotechnology and Nanomedicine, College of Pharmacy, College of Medicine, Dorothy M. Davis Heart and Lung Research Institute, and James Comprehensive Cancer Center, The Ohio State University, Columbus, OH 43210 USA; 15 CRISPR Gene Targeting Core, Taipei Medical University, Taipei, 110 Taiwan; 16 Department of Biological Science & Technology, College of Life Sciences, China Medical University, Taichung, Taiwan

**Keywords:** Anti-HER2 therapy responder, Breast cancer, Histamine N-methyltransferase, H-cell phenotype, Nuclear translocation

## Abstract

**Background:**

Up to 23% of breast cancer patients recurred within a decade after trastuzumab treatment. Conversely, one trial found that patients with low HER2 expression and metastatic breast cancer had a positive response to trastuzumab-deruxtecan (T-Dxd). This indicates that relying solely on HER2 as a single diagnostic marker to predict the efficacy of anti-HER2 drugs is insufficient. This study highlights the interaction between histamine N-methyltransferase (HNMT) and HER2 as an adjunct predictor for trastuzumab response. Furthermore, modulation of HER2 expression by HNMT may explain why those with low HER2 expression still respond to T-Dxd.

**Methods:**

We investigated the impact of HNMT protein expression on the efficacy of anti-HER2 therapy in both in vivo and ex vivo models of patient-derived xenografts and cell line-derived xenografts. Our analysis included Förster resonance energy transfer (FRET) to assess the interaction strength between HNMT and HER2 proteins in trastuzumab-resistant and sensitive tumor tissues. Additionally, we used fluorescence lifetime imaging microscopy (FLIM), cleaved luciferase, and immunoprecipitation to study the interaction dynamics of HNMT and HER2. Furthermore, we evaluated the influence of HNMT activity on the binding of anti-HER2 antibodies to their targets through flow cytometry. We also observed the nuclear translocation of HNMT/HER2-ICD cells using fluorescent double staining and DeltaVision microscopy. Finally, ChIP sequencing was employed to identify target genes affected by the HNMT/HER2-ICD complex.

**Results:**

This study highlights HNMT as a potential auxiliary biomarker for diagnosing HER2 + breast cancer. FRET analysis demonstrated a significant interaction between HNMT and HER2 protein in trastuzumab-sensitive tumor tissue (*n* = 50), suggesting the potential of HNMT as a predictor of treatment response. Mechanistic studies revealed that the interaction between HNMT and HER2 contributes to increased HER2 protein expression at the transcriptional level, thereby impacting the efficacy of anti-HER2 therapy. Furthermore, a subset of triple-negative breast cancers characterized by HNMT overexpression was found to be sensitive to HER2 antibody–drug conjugates such as T-Dxd.

**Conclusions:**

These findings offer crucial insights for clinicians evaluating candidates for anti-HER2 therapy, especially for HER2-low breast cancer patients who could gain from T-Dxd treatment. Identifying HNMT expression could help clinicians pinpoint patients who would benefit from anti-HER2 therapy.

**Supplementary Information:**

The online version contains supplementary material available at 10.1186/s40364-024-00715-5.

## Introduction

Breast cancer (BC) is the most commonly diagnosed cancer and has a high mortality rate (15.0 per 100,000 people) in transition countries [1]. Of these, 14% to 30% of primary human BCs are of the human epidermal growth factor receptor 2 (HER2)-positive (HER2 +) subtype [2]. This subtype exhibits more aggressive behavior and has a worse prognosis if left untreated [3, 4]. Trastuzumab therapy improves outcomes and prolongs survival in patients with HER2 + BC and is now the standard of care for adjuvant and metastatic therapy [5–7]. The current classification of anti-HER2 targeted therapy is as follows: 1. Antibody drugs: trastuzumab and pertuzumab, 2. Small molecule inhibitors: lapatinib and neratinib, 3. Antibody–drug conjugates (ADCs): trastuzumab emtansine (T-DM1) and trastuzumab deruxtecan (T-Dxd), 4. Others include HER2 peptide vaccines and CAR-T-cell therapy [8].

New avenues have emerged in HER2/RTK treatment research, offering the potential for further exploration. According to a recent study, a novel dual-function approach, Phosphorylation targeting chimeras (PhosTACs), has been developed to achieve targeted protein dephosphorylation (TPDephos). The concept combines the inhibitory effect of receptor tyrosine kinase inhibitors (RTKIs) with active phosphatase dephosphorylation to achieve dual kinase inhibition. An example of this is PhosTAC (GePhos1), which has been shown to modulate HER1 signaling, leading to apoptosis and cytotoxicity through the induction of HER1 dephosphorylation [9]. Based on the concept of proximal protein degradation, proteolysis targeting chimeras (PROTACs) function through a three-enzyme ubiquitination system consisting of E1, E2, and E3 ligases. This intricate system orchestrates the targeted degradation of specific proteins, offering promising potential for therapeutic interventions [10]. Nanoscale hybrid proteolytic targeting chimeras (GNCTACs) leverage gold nanoclusters (GNCs) as a platform to facilitate the linkage of HER2 targeting peptides and cereblon, thereby enhancing the process of HER2 endocytosis and promoting protein degradation. This mechanism ultimately augments the ratio of protein degradation, leading to the promotion of programmed cell death [11].

Anti-HER2 targeted drugs at the initial stage of treatment can improve the survival rate of HER2 + BC patients [12]; however, after long-term follow-up, drug resistance in HER2 + tumors is still inevitable. Up to 23% of patients with early-stage HER2 + treated with adjuvant chemotherapy and trastuzumab relapse within ten years [13, 14]. The APHINITY study showed that 10% of patients treated with trastuzumab relapse within six years, and if dual-targeted therapy with pertuzumab and trastuzumab were used, the recurrence rate would decrease to 7% within five years [15]. The NeoALTTO study revealed that 22% of patients relapsed within three years of treatment with lapatinib alone, and even with lapatinib combined with trastuzumab dual-target therapy, the recurrence rate was still as high as 17% [16]. Compared with antibody drugs, T-Dxd is the newest ADC drug for HER2-low BC patients. T-DM1 can significantly prolong the survival time of patients with HER2 + metastases and reduce the risk of recurrence; however, T-DM1 therapy still has a 10% distant metastasis recurrence rate [17].

The clinical and biological heterogeneity of HER2 + BC complicates the identification of responders from initial breast tumor biopsies. Many HER2 + patients diagnosed by current HER2 assessment do not have an expected good treatment response to anti-HER2 therapies [18, 19]. The use of genomic and proteomic analyses to predict the benefit of trastuzumab in adjuvant treatment in BC cohorts is a recent trend [20–23]. Therefore, a second auxiliary diagnostic molecular marker is necessary to identify patients who respond to anti-HER2 therapy early. A literature search revealed that *histamine N-methyltransferase* (*HNMT*) mRNA was highly expressed in 83% of breast cancer cell lines (BCLs) (31 BCLs), especially HER2 + BCL, but not in the normal breast epithelial cell line MCF-10A [24]. This study showed that HNMT protein overexpression was preferentially detected in HER2 + BCLs (*n* = 8). The Cancer Genome Atlas (TCGA) and Cancer Cell Line Encyclopedia (CCLE, Expression Public 22Q4) databases also showed that *HNMT* mRNA was detected higher in a HER2 + subgroup. This study shows that HNMT is another valuable poor prognostic indicator besides the HER2 protein [4].

The function of a protein (signal transduction, transport, and metabolism) depends on structural changes and reactions resulting from interactions with other proteins. Abnormal Protein–protein interaction (PPI) is known to be associated with the development of cancer cells [25]. Therefore, clarifying PPIs can help develop strategies to treat the disease. Studies indicate that PPIs between HER2 and other proteins (except those in the HER family) affect HER2 signaling and subsequent therapeutic effects of HER2 inhibitors. CUB domain-containing protein 1 forms a PPI with HER2 through its intracellular domain and promotes HER2-c-SRC crosstalk interaction, leading to trastuzumab resistance [26]. HER2 interacts with nuclear factor erythroid 2-related factor 2 (NRF2) and initiates induction of NRF2 transcription, detoxification, and drug efflux proteins that confer drug resistance to cancer cells [27]. Heat shock protein 90 (HSP90) can bind to multiple proteins, such as HER2 (full-length and p95HER2), and can regulate HER2 activity, but most patients will have recurrence and metastasis and eventually become drug-resistant [28]. The recently developed HSP90 inhibitor (NCT-547) effectively promotes the degradation of full-length HER2 and the truncation of p95HER2 while weakening the heterodimerization of HER2 family members. Therefore, these previous findings inspired our study of HNMT-HER2 interaction.

The physiological function of normal intracellular HNMT is a cytoplasmic enzyme that inactivates intracellular histamine by catalyzing the transfer of a methyl group from S-adenosyl-L-methionine to histamine [29]. Upon growth factor receptor-mediated stimulation, cytoplasmic HNMT translocates to the plasma membrane and binds to the organic cation transporter, a histamine transporter [30]. Epidermal growth factor (EGF)-induced HNMT translocation to the plasma membrane was also observed in this study. This study further demonstrated that HNMT promotes the formation and nuclear translocation of the intracellular domain (ICD) of HER2 (HER2-ICD) to activate the transcription of the HER2 protein in BC cells. A similar story in our previous study reported that the nuclear p185HER2 is recruited to the promoters/enhancers of target genes and acts as a transcriptional regulator [31]. Another study observed that the HER2-ICD in triple-negative BC (TNBC) cells promotes cancer cell proliferation [32]. In this study, we demonstrated that forced HNMT overexpression significantly induced HER2 protein levels in HER2 + and triple-negative BCLs, whereas inhibition of HNMT expression decreased HER2 protein levels in HER2 + BCLs. However, the knockdown of HER2 protein expression in HER2 + BCLs did not affect the protein level of HNMT. These results suggest that HNMT is an upstream regulator of HER2 protein expression. Similar studies support our hypothesis, such as caveolin 1, a HER2-interacting protein that sensitizes HER2 + cancer cells to HER2-targeted drugs [33].

According to research, early HER2 + BC patients receiving adjuvant chemotherapy and trastuzumab treatment have a high risk of recurrence within ten years, with up to 23% of patients experiencing recurrence. This indicates that the traditional single diagnostic biomarker (HER2) for screening patients for trastuzumab treatment and preventing recurrence still needs improvement. A significant breakthrough of this study is the demonstration of HNMT as an effective adjunct biomarker for predicting the efficacy of anti-HER2 treatment in HER2 + BC patients. Further molecular mechanism studies have confirmed that HNMT can act as a transcriptional regulator of the HER2 protein, converting cold tumors (HER2-negative) into hot tumors (HER2 +). In animal models, increasing HNMT expression in TNBC tumors enhanced sensitivity to anti-HER2 therapy (trastuzumab). Therefore, in addition to predicting the therapeutic effect of anti-HER2 drugs in HER2 + patients, targeting the subgroup of patients with high HNMT expression in TNBC tissues for anti-HER2 drug treatment (such as T-Dxd) may serve as a new option for therapy. These research findings are expected to significantly improve BC patient's treatment success and survival rates.

## Methods

### Cell lines

Cell culture reagents, such as media, serum, antibiotics, and trypsin–EDTA, were procured from Invitrogen or Gibco (ThermoFisher Scientific) unless stated otherwise. The UACC812, HCC202, HCC1954, UACC893, SKBR3, BT-474, AU565, HCC1937, BT-549, Hs-578 T, MDA-MB-231, MDA-MB-436, MDA-MB-453, HCC1395, HCC38, BT-20, MDA-MB-361, MCF-7, T47D, ZR-75–1, and MCF-10A cell lines were obtained from the American Type Culture Collection (ATCC) and underwent rigorous testing and ATCC certification. All human cell lines have been authenticated using STR profiling within the last three years. All experiments were performed with mycoplasma-free cells. MCF-10A, a nontumorigenic human mammary epithelial cell line, and all other cell lines were cultured following previously published protocols [34, 35].

### Human patient samples

All 766 breast tumor samples were acquired from female patients who underwent surgery at Taipei Medical University Hospital with Institutional Review Board (IRB number: CRC-14–10-01) approval. Written informed consent was obtained from all patients, and the research adhered to ethical standards, including the 1964 Declaration of Helsinki. The data release complied with the Human Tissue Act for pseudo-anonymization. Each sample contained more than 80% tumor tissue, as confirmed by histological examination and clinical categorization. All the samples were preserved in liquid nitrogen.

### Chemicals and antibodies

The reagents used in the study were obtained from various sources. G418 (4131) and neuregulin-1 (NRG1, 396HB) were purchased from R&D Systems. Doxycycline (DOX, D9891), cycloheximide (CHX, 01810), and MW167 (Calbiochem, 565755, a γ-secretase inhibitor) were acquired from Sigma‒Aldrich. EGF (PHG0311) and hygromycin B (106870110) were obtained from ThermoFisher Scientific. DAPT (2634, a γ-secretase inhibitor) was procured from TOCRIS, and VivoGlo™ Luciferin (P1043) was sourced from Promega. All reagents were used according to the manufacturer's instructions to ensure the accuracy and reproducibility of the experimental results.

The experiments utilized commercial antibodies obtained from various suppliers for Western blotting, immunofluorescence (IF) staining, immunoprecipitation (IP), and immunohistochemistry (IHC) staining, following the recommended protocols provided by the respective manufacturers. The antibodies listed below are specific for the following antigens: HNMT (H00003176-M12, Abnova; PA5-11,499, ThermoFisher Scientific), HER1 (8339, Cell Signaling), HER2 (3B5) (sc-284, Santa Cruz), trastuzumab (Roche), T-Dxd (Daiichi-Sankyo/AstraZeneca), HER3 (8339, Cell Signaling), HER4 (8339, Cell Signaling), α-Tubulin (GTX628802, GeneTex), pHER2 (Tyr1221/2) (2243, Cell Signaling), pHER2 (Tyr1248) (2247, Cell Signaling), p-AKT (4060, Cell Signaling), T-AKT (Santa Cruz, sc-265943), PS1 (5887, Cell Signaling), GAPDH (ab8245, Abcam), NuP98 (ab124980, Abcam), Lamin A/C (NB100-14457, Novus) and normal rabbit IgG (Sigma‒Aldrich). The experiment utilized a variety of secondary antibodies for different applications. Anti-rabbit IgG-HRP (GTX213110-01, GeneTex) and anti-mouse IgG-HRP (AP124P, Sigma‒Aldrich) antibodies were employed for western blotting. For IHC, anti-human HRP polymer (ab214883, Abcam), anti-mouse HRP polymer (VC001, R&D Systems), and anti-rabbit HRP polymer (VC003, R&D Systems) antibodies were used. Additionally, for IF staining, a range of antibodies from Jackson Immunological Research Laboratories were utilized, including anti-human FITC (109–096-003), anti-mouse rhodamine (715–025-151), anti-rabbit FITC (111–095-003), and anti-rabbit rhodamine (111–025-003) antibodies.

### RNA extraction, reverse transcription (RT), and real-time quantitative PCR

Total RNA was isolated from BC normal and tumor tissues with TRIzol reagent (ThermoFisher Scientific) following our previously published protocol [34]. Subsequently, complementary DNA was produced using a reverse transcriptase kit (Toyobo). *HNMT* mRNA fluorescence intensity was measured with a Roche LightCycler version 4.0 built-in software and then normalized to *GUS* mRNA expression. The specific sequences of primers (Additional file 1:Table S1) used for *HNMT* and *GUS* are available for reference.

### Construction of recombinant plasmids

In this study, all primers utilized for amplifying the chosen sequences are listed in the supplementary file (Additional file 1:Table S1). Additionally, all plasmid constructs were validated through DNA sequencing analysis to confirm their identities. The siRNA plasmids, designed with powerful sequences for RNA interference against specific genes using Oligoengine 2.0, were meticulously integrated into the pSUPER.retro.neo-GFP plasmids from Oligoengine after cleavage with *BglII* and *HindIII*, ensuring precise integration for targeted RNAi effects. The knocked-out (KO) plasmids, designed with sgRNA sequences for KO against HNMT or HER1 genes using ChopChop, were integrated into the sgRNA pAll-Cas9 pPuro plasmid after cleavage with BbsI, ensuring precise integration for targeted KO effects. PCR was used to amplify specific HNMT sequences that were subsequently cloned and inserted into different plasmids. These plasmids included the *HindIII* and *XbaI* cleaved pcDNA5/TO expression vector (V103320, ThermoFisher Scientific), the *SalI* and *BamHI* cleaved pcDNA3.1-ZsYellow plasmid, and the *EcoRV* and *XbaI*-cut pcDNA3.1-N-Luc plasmid, resulting in the creation of the specified vectors. Similarly, for the HER2 expression plasmids, selected HER2 sequences were amplified using PCR and cloned and inserted into different plasmids. Specifically, the fragment was inserted into the *NheI-* and *HindIII*-cleaved pcDNA3.1-AmCyan plasmid, as well as the *XhoI-* and *XbaI*-cleaved pcDNA3.1-C-Luc plasmid, resulting in the generation of the indicated plasmids. In addition, 4X HNMT binding site (HBS) reporter plasmids were generated by synthesizing four repeats of identified binding site sequences from ChIP-sequencing data and inserting them into *NheI-* and *XhoI*-cut pGL3 Luciferase (Luc) reporter plasmids (Promega) to create the specified plasmids. The specified plasmids were transfected into 2 × 10^6^ cells using 4 μg of plasmid and the Neon™ transfection system (MPK10096, ThermoFisher Scientific).

### Generation of stable cells

Following transfection, the cells were grown for 3 days and then selected with G418 for cells harboring pSUPER and pcDNA3.1-Luc plasmids, with hygromycin B for cells containing pcDNA5/TO vectors, and with puromycin (0.75 μg/mL) for MDA-MB-231 cells containing the sgRNA pAll-Cas9.P puro plasmid. The selection lasted 14 days to isolate and enhance the transfected cells.

### Cell growth

Upon plating either five thousand transiently transfected cells (231-Wt, 231-Sc, 231-Si) or two thousand transiently transfected cells (231-Wt, 231-Vc, 231-HN) in 96-well plates, the cells were observed for two days. Subsequently, cell viability was evaluated using MTT relative to untreated controls.

### Cell viability

In a series of experiments, two thousand cells (comprising 231-Wt, 231-Vc, 231-HN, and HCC1954) were seeded in 96-well plates and exposed to varying concentrations of T-Dxd (ranging from 0 to 50 μg/mL) for six days. Cell viability was assessed using MTT relative to untreated controls, and IC_50_ values were determined utilizing non-linear regression analysis with GraphPad Prism 8 software. Additionally, five thousand cells (including AU565, SKBR3, BT-474, and MDAMB-231) along with one thousand cells (MCF-10A) were plated in 96-well plates and treated with different concentrations of SKF-91488 (ranging from 0 to 100 μM) for two days. Subsequently, cell viability was measured relative to untreated controls using MTT.

### Western blot analysis

After cell lysis at 4°C using gold lysis buffer, the protein concentration was determined using the Bradford assay (Bio-Rad) supplemented with protease inhibitors. Next, 50 μg of protein lysates were subjected to SDS‒PAGE (10‒12%), transferred to PVDF membranes, and probed with a specific primary antibody for 2 h at room temperature. HRP-conjugated secondary antibodies were followed, and visualization was performed using an enhanced chemiluminescence substrate (GE Healthcare). The uncropped blot scans are available in the source data file.

### Cycloheximide (CHX) chase assay

Cells were treated with CHX (20 μg/mL) (Sigma) for various periods (0, 24, and 48 h). Cells were pretreated with γ-secretase inhibitors (10 μM DAPT and 10 μM MW-167) for 24 h to inhibit γ-secretase activity. Each cell lysate (50 μg) was analyzed by Western blotting with the corresponding antibody.

### Wound healing assay

The cells were plated at a density of 1 × 10^4^ cells/well in μ-insert wells (Ibidi GmbH). Following removing the μ-insert, a 0.5 mm linear wound was created. Time-lapse images were taken using a Leica DMI 4000B Microscope Imaging System (Leica), and the wound area was calculated at specific time points relative to the 0-h time point using ImageJ software.

### Colony formation assay

The soft agar assay was performed according to protocols detailed in our earlier publications [34, 36]. The cells were incubated at 37°C, and colony development was observed over 21 days using a Leica DMI 4000B microscope imaging system.

### Invasion assay

The study involved seeding 10,000 cells in Transwell^®^ inserts coated with BioCoat™ Matrigel matrix at a fourfold dilution. After a 36-h incubation period, the invading cells were identified by staining with 0.5% crystal violet reagent containing 10% formaldehyde for 10 min. The stained cells were then imaged using a Leica DMI 4000B microscope (Leica) for further analysis [37].

### Doxycycline(DOX)-inducible HNMT expression in the T-REx™ system

Following transfection, the cells were allowed to recover for 48 h before being treated with 5 μg/mL DOX for different durations up to 48 h to induce HNMT expression.

### Membrane and cytoplasmic protein extraction

The cells were lysed in homogenization buffer (20 mM Tris (pH 7.5), 5 mM EGTA, and 20 mM EDTA) supplemented with protease inhibitors and then disrupted by gently pipetting 30 times with a syringe on ice. Subsequently, the cell lysate was centrifuged at 12,000 rpm for 10 min at 4°C, and the resulting supernatant was transferred to a new microcentrifuge tube to obtain the cytoplasmic fraction. The following two washes of the pellet fractions were lysed in golden lysis buffer and further disrupted every 5 min for 45 min using a vortex mixer. After centrifugation at 12,000 rpm for 30 min at 4°C, the supernatant was collected as the membrane fraction. Finally, the protein levels in the cytoplasmic and membrane fractions were analyzed by western blotting.

### Immunoprecipitation (IP)

Cell lysates were extracted from the specified cells using a lysis buffer. Following our previous report [35], 500 μg of protein lysates were combined and incubated with primary and normal rabbit IgG antibodies. The resulting mixture was then treated with 40 μL of protein G-conjugated agarose beads (16–266, Sigma‒Aldrich) for 1 h at 4°C, followed by four PBS washes and analysis by western blotting. Nonspecific IgG served as the negative control.

### Flow cytometric analysis

The cells were fixed with 4% paraformaldehyde for 15 min and then incubated with varying concentrations of trastuzumab and anti-HNMT antibody overnight at 4°C. Following washing with ice-cold 1 × PBS, the cells were incubated with anti-human FITC and anti-mouse rhodamine antibodies for 1 h at 4°C before analysis using a flow cytometer (Sony SA3800, SONY). This assay was conducted three times with duplicate samples to ensure reproducibility.

In addition, the cells were treated with 10 μg/mL T-Dxd for various durations and then subjected to a similar assay using a flow cytometer (CytoFLEX, Beckman Coulter). This procedure was repeated three times with duplicate samples to validate the findings.

### Laser capture microdissection (LCM)

The breast tumor samples were embedded in optimal cutting temperature compound, frozen in dry ice-cold 2-methylbutane (~ –60 °C), and then cut into 10 μm thick sections for LCM (Leica CM3050S). The sections were fixed, rehydrated, stained, dehydrated, and cleared before LCM. LCM was performed on HER2 + and TNBC tumor sections using the PixCell II system. Total RNA was extracted from LCM samples using the MicroRNA Isolation Kit with modifications, followed by real-time quantitative RT‒PCR analysis as described in our previous publication [34].

### Luciferase(Luc) reporter assay

The cells were lysed with Reporter Lysis Buffer (Promega) and stored at -20°C overnight. Firefly and *Renilla* luc signal measurements were performed using a Dual-Luciferase Reporter Assay Kit (#E1960; Promega). Total luc activity was quantified using a HIDEX Chameleon Microplate Reader (BioTek). The *Renilla* luc construct pRL-TK served as an internal control. Relative luc activity was determined by normalization to *Renilla* luc activity within the same cell lysate. All experiments were replicated twice in three independent experiments.

### Immunohistochemistry (IHC) and IHC double staining

The IHC staining protocol utilized in this study closely followed the methodology outlined in our previous publication [34]. Briefly, PDX, CDX, BC patient tumors, and tissue microarrays (TMAs, IRB number N201812005) were promptly fixed in 10% buffered formalin solution for up to 24 h at room temperature after excision and then dehydrated and embedded in paraffin (FFPE). IHC and IHC double staining were conducted on 4-μm-thick FFPE tissue sections. Slides were deparaffinized with xylene and rehydrated with decreasing ethanol concentrations in water. For IHC staining, the FFPE tissue sections were subjected to a 2 h incubation with the primary antibody, followed by a 1-h incubation with the secondary antibody, and ultimately visualized using diaminobenzidine (DAB) solution (K3468, Dako). Trastuzumab targeting p185HER2 was visualized with a DAB (brown) solution. Secondary IHC staining with antibodies against HNMT and HER2-ICD was visualized using Vina Green™ Chromogen solution (green, BRR807AH, Biocare Medical). Anti-HNMT and anti-T-Dxd stained IHC images were quantified using ImageJ software with the IHC Profiler plug-in.

### Histo-scoring

After careful examination by at least two pathologists, the HNMT and HER2 IHC results were meticulously evaluated for staining intensity and the proportion of positively stained tumor cells. The histo-score was calculated using the formula Histo-score = i × p, where "i" represents the staining intensity on a scale from 0 to 3 + and "p" denotes the percentage of stained tumor cells. The established cutoff value was determined based on the mean HNMT tissue score of 114%. A score exceeding 114% indicated high HNMT expression.

### Chromatin immunoprecipitation (ChIP) assay

The cells were treated with EGF (100 ng/mL) for 24 h, fixed in 1% formaldehyde, and quenched with glycine (2.5 μM) for 5 min. After sonication, the supernatant was combined with protein G beads (16–266, Sigma‒Aldrich) and antibodies against HNMT and HER2 or normal rabbit IgG. The DNA‒protein complexes were eluted, and the enriched DNA was used for real-time PCR assays targeting the HER2 promoter. The fluorescence intensity was measured using a Roche LightCycler version 4.0, and the specific sequences of primers used can be found in the supplementary file (Additional file 1:Table S1).

### ChIP-sequencing analysis

Proteins for ChIP were obtained from SKBR3 BC cells after 72 h of treatment with epidermal growth factor (EGF). H_2_O-treated cells were used as controls. The DNA samples were then subjected to ChIP-sequencing using the MISSION BIOTECH platform. Genomic DNA was processed, and template preparation and chip loading were carried out per the Ion Chef user guide. Sequencing was performed using the Ion-Sequencing IC 200 Kit on the Personal Genome Machine™ (PGMTM) sequencer, followed by alignment to the human reference genome using Torrent Suite v5.0 and Genomics Suite Software for peak calling.

### Immunofluorescence (IF) staining for Förster resonance energy transfer (FRET) and deconvolution microscopy

IF staining was conducted using our previous protocol with minor modifications [35]. The samples were fixed in 4% paraformaldehyde for 15 min, blocked with 3% FBS, 1% BSA, 1% gelatin, and 0.5% Tween-20 in PBS for 30 min, and then incubated with primary antibodies for 2 h, followed by incubation with secondary antibodies for 1 h at room temperature. Similar procedures were applied to ex vivo tumor samples, including xenografted tumor tissues. The mounted samples were examined using a Leica TCS SP5 Confocal Spectroscopic Imaging System or a GE Healthcare DeltaVision Personal Deconvolution Microscope.

Quantitative colocalization measurements were conducted using the FRET AB Software of the Leica TCS SP5 Confocal Spectral Microscopy Imaging System (Leica). The acceptor bleaching FRET method followed the manufacturer's instructions, as outlined in the FRET Wizards in the Leica Application Suite. Further details of this method can be found in our previous publication [35]. The FRET efficiency was determined using FRET_eff_ = (D_post_-D_pre_)/D_post_. The analysis involved calculating the FRET efficiency in a minimum of 15 cells. A significance level of *P* < 0.05 was applied to the results. Deconvolution fluorescence images were processed using Volocity™ 6.3 software (Quorum Technologies Inc). Quantification of fluorescent images stained with anti-HNMT/anti-HER2-ICD or anti-HNMT/anti-trastuzumab using the Colocalization_Finder plug-in for ImageJ software.

### Uptake of T-Dxd

231-Wt, 231-Vc, 231-HN, and HCC1954 cells were seeded onto coverslips and treated with T-Dxd (10 µg/mL) for 16 h. The cells were then fixed in 4% paraformaldehyde, blocked with a solution of 3% FBS, 1% BSA, 1% gelatin, and 0.5% Tween-20 in PBS, and incubated with a secondary antibody and LysoTracker^TM^ Red DND-99 (L5728, at a 100-fold dilution) for 1 h. Afterward, the cells were stained with Hoechst for 10 min at room temperature. The samples were mounted using VECTASHIELD^®^ Antifade Mounting Medium from Vector Labs and analyzed using a Leica TCS SP5 confocal spectral imaging system.

### Live-cell confocal microscopy and fluorescence lifetime imaging microscopy (FLIM)

After transfection, the cells were cultured in a live-cell system connected to a TCS SP5 confocal spectral microscope (Leica). The FLIM system utilized time-correlated single-photon counting to record the AmCyan lifetime over a 10-min period. Images were generated by analyzing pixel-by-pixel fluorescence decays to determine the average lifetime. The FLIM-FRET efficiency was computed using the formula E = 1-(τ_DA_/τ_D_), where τ_DA_ is the lifetime of the donor in the presence of the acceptor, and τD is the lifetime of the donor in the absence of the acceptor.

### Validation of patient-derived xenograft(PDX) tumors

We purchased PDX tumor-bearing mice (JAX 4659679, J000100674 (PDX-M1), and J000111056 (PDX-M2)) from Jackson Laboratories (JAX, 005557, USA). After the PDX tumor-bearing mice with the fourth-generation PDX tumor tissue were sacrificed, the tumor tissues were removed and transplanted subcutaneously into NOD.Cg-*Prkdc*^*scid*^* Il2rg*^*tm1Wjl*^/SzJ (NSG) mice for further research. We performed RNA sequencing analysis to confirm the concordance of genetic profiles between PDX tumors from JAX (passage 4) and PDX tumors from our laboratory (passage 6). A heat map comparing JAX's RNA sequencing data with data from our laboratory is provided for reference (Additional file 11: Fig. S11). In addition, our previous study evaluated the response to trastuzumab in two PDX mouse models. Details can be found in the related article [38].

### RNA sequencing and RNA-seq data analyses

Total RNA from passage 6 PDX tumors and MDA-MB-231 (231-Vc and 231-HN with/without EGF treatment, 100 ng/mL for 24 h) cells were purified using the TruSeq Stranded mRNA Library Prep Kit (Illumina, USA) according to the manufacturer's recommendations. Each library was sequenced using the Illumina NovaSeq6000 platform with 150 bp paired-end reads (Genomics, BioSci & Tech Co, Taiwan). The Trimmomatic program (version 0.39) was used to remove low-quality bases (base quality ≤ 5) and sequences from adapters in RNA-seq raw data. Filtered reads were aligned to the reference genome (GRCh37) using Bowtie2 (version 2.3.4.1). Gene (DEG) (version 1.16.0). The raw data have been uploaded to the Genome Sequence Archive (GEO), and the link is in the data availability statement.

### Animal model of xenograft tumor formation and in vivo split-luc assay

The animals for the study were obtained from the National Defense Medical Center in Taipei, Taiwan, and were cared for following the LAC-101–0064 protocol. Subsequently, 1 × 10^6^ stable cells were injected into 4-week-old nude mice bearing HNMT KO xenografts (*n* = 5), and 4-week-old NOD mice bearing HNMT-overexpressing xenografts were generated. The efficacy of trastuzumab (15 mg/kg, IV injection, twice weekly) in CB17-Prkdc SCID/J (NOD-SCID) mice (*n* = 3), stable cells for trastuzumab (15 mg/kg, IV injection, twice weekly) treatment of 4-week-old nude mice (*n* = 3), and T-Dxd (4 mg/kg, IV injection, once weekly) treatment of 4-week-old NOD–SCID–IL2Rγ^null^ (NSG) mice (*n* = 3) were studied. Additionally, cells were implanted into female NOD-SCID mice for the metastasis assay and injected into 4-week-old nude mice for the in vivo split-luc assay (*n* = 2). Tumor sizes were monitored following the formula: tumor volume (cm^3^) = ½ × L × W^2^.

### In vivo TNBC patient-derived xenograft (for PDX) mouse model

The NSG mice and PDX-bearing mice (J000100674, model-1, PDX-M1; J000111056, model-2, PDX-M2) were obtained from Jackson Laboratories (JAX#4,659,679). Both PDX-M1 and PDX-M2 originated from female patients (005557), and age details were not provided. The patients were non-Hispanic or Latino, with breast tumors classified as Grade III (AJCC IIIc). Fourth-generation PDX tumor tissues from Jackson Laboratories were transplanted into NSG mice in our laboratory for further research.

The mice in this study were handled and maintained according to the Animal Resource Center guidelines and approved by the Laboratory Animal Care and Use Committee at the National Defense Medical Center (IACUC protocol IACUC-20–054). Using an orthotopic approach, the PDX model involved implanting tumor tissues into NSG mice. PDX-bearing mice (6th generation) were divided into groups of 5 mice each and received 8 trastuzumab injections at a dosage of 15 mg/kg twice weekly.

Tumor volume was assessed over 31 days, with measurements taken every two days using the formula = ½ × L × W^2^. The control group received normal saline treatment. RNA sequencing analysis compared 4th-generation JAX PDX tumors with 6th-generation tumors from our laboratory to ensure genetic consistency. Additional file 11: Fig. S11 includes a heatmap comparing RNA sequencing data from JAX with our laboratory's data.

### Establishment of primary cultured cells

The xenograft tumor or lung tissue was carefully dissected and placed in Petri dishes for 2–5 days. After removal, G418 or hygromycin B was added for 14 days to select stable cells, ultimately yielding primary cells.

### Statistical analysis

The data are presented as the mean ± SEM from > 3 independent experimental replicates. *HNMT* mRNA expression levels in normal and tumor tissues from BC patients were compared using the Mann‒Whitney U test. Pathology, N status, stage, and grade data were analyzed using one-way ANOVA. Differences between groups were compared using an unpaired Student's t-test. Nonlinear regression and Pearson correlation analyses were conducted for histo-score calculation and *P* value determination. Statistical comparisons were performed using SigmaPlot graphing software and SPSS v.11.0.0. *P* ≤ 0.05 was considered to indicate statistical significance.

## Results

### HNMT overexpression in HER2 + breast tumor patients with poor clinical outcomes

**Fig. 1:**
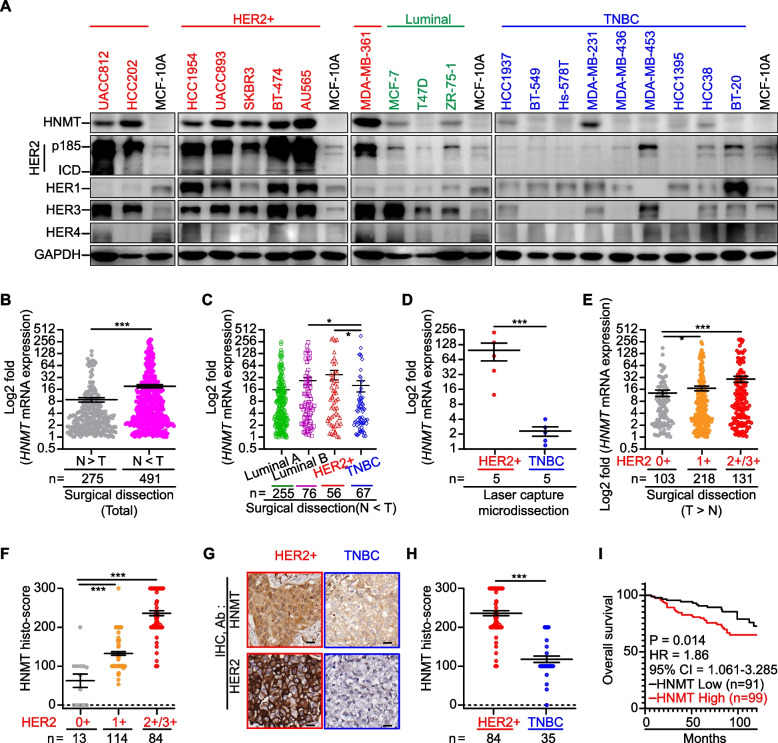
HNMT expression in human BC patients and cell lines. **A** Western blot analysis confirmed the presence of HNMT and HER receptors in various cell types. **B-E** Subsequent quantitative real-time PCR analysis revealed differential *HNMT* mRNA expression in paired normal and tumor tissues from total BC patients (**B**) and in tissues from different subtype patients (**C**). Additionally, laser capture microdissection of cells from HER2 + and TNBC patients revealed distinct levels of *HNMT* mRNA expression (**D**). The fold change in *HNMT* mRNA expression according to HER2 status (**E**). **F** Histological scoring of HNMT protein levels by IHC staining in breast tumors from BC patients indicated a correlation with increased HER2 status. **G-H** Representative IHC images (**G**) and statistical analysis (**H**) demonstrate the HNMT protein's differential expression in mammary tumors from HER2 + and TNBC patients. **I** Kaplan‒Meier analysis revealed a potential association between HNMT protein expression and overall survival in BC patients. The data are presented as the mean ± SE, and statistical analysis was performed using a two-tailed unpaired Student's t-test (for laser capture microdissection data), a two-tailed Mann‒Whitney U test (for mRNA expression and histological scoring data), and a log-rank test (for survival analysis). Significance levels are denoted as *P < 0.05, **P < 0.01, and ***P < 0.001

A previous study [24] showed that *HNMT* mRNA is highly expressed in 31 different types of BCLs, especially in HER2 + BCLs. Therefore, we asked whether HNMT expression might be related to HER2 expression and, if so, whether this relationship might be related to BC malignancy. We validated HNMT protein expression levels in BCLs and human BC tumor tissues with known clinical parameters to test this observation (Fig. 1A and Table 1). The results (Fig. 1A) showed that higher levels of HNMT protein were detected in HER2 + BCLs than in TNBC, luminal BCLs, and normal epithelial cells. However, luminal BCLs do not express higher levels of HNMT, and we believe that HNMT expression is independent of the ERα protein. The results from the CCLE database showed that the mRNA expression level of *HNMT* was moderately correlated with *HER2* in BCLs (*n* = 54, R^2^ = 0.4126, Additional file 1: Fig. S1A). Furthermore, subgroup-based data analysis showed that *HNMT* mRNA expression was higher in HER2 + BCLs than in TNBC BCLs (*P* = 0.008, Additional file 1: Fig. S1B). The analysis results from the TCGA database also showed that *HNMT* mRNA expression was higher in the tumor tissues of HER2 + BC patients than in TNBC BC patients (*P* < 0.001, Additional file 1: Fig. S1C). Real-time PCR was used to determine *HNMT* mRNA levels in 766 human tissue sample pairs (tumor vs. normal) (Fig. 1B and Additional file 1: Fig. S1D). The data shown in Fig. 1b were divided into two groups (N > T, *n* = 275, vs. T > N, *n* = 491; N = normal, T = tumor) according to the *HNMT* mRNA expression pattern. In the T > N ​​group (Fig. 1B), *HNMT* mRNA expression in tumor cells was significantly higher (20.58-fold) than that in normal cells. In this T > N group, we have presented the *HNMT* mRNA expression levels of patients with Luminal A, Luminal B, HER2 + , and TNBC BC subtypes, as shown in Fig. 1C. Our analysis revealed that there were no significant differences in *HNMT* mRNA expression between Luminal A and Luminal B BC patients (*P* = 0.0949), as well as between Luminal A and HER2 + BC patients (*P* = 0.1274). However, we observed a significant difference in *HNMT* mRNA expression levels between Luminal B (ER + /HER2 +) and TNBC subtypes (*P* = 0.0281), as well as between HER2 + and TNBC subtypes (*P* = 0.0325) of BC. These findings highlight the potential relevance of *HNMT* mRNA expression in HER2 + BC subtypes and may have implications for targeted therapeutic strategies. To validate the findings in Fig. 1C, we analyzed *HNMT* mRNA expression utilizing samples obtained through LCM. Our investigation yielded consistent results, further supporting the initial observation (*n* = 5 per group, Fig. 1D and Additional file 1: Fig. S1E). The mRNA levels of *HNMT* in the T > N group (fold change relative to that in normal tissue) were positively correlated with HER2 protein overexpression (IHC score > 2 +) in BC tumors (Fig. 1E).

**Table 1:** Demographic and clinical characteristics associated with the fold differences in *HNMT* mRNA expression between paired tumor and normal tissue samples

Parameter	N > T group (#)			T > N group (#)		
	n	Mean (§) ± SE	*P* value	n	Mean (§) ± SE	*P* value
**Age**			0.414			0.014
< 55 years	139	9.10 ± 18.84		243	16.28 ± 35.95	
≥ 55 years	123	8.28 ± 18.59		236	23.95 ± 49.13	
**Pathology**			0.779			0.038
Ductal Carcinoma In Situ	8	5.42 ± 4.34		21	4.63 ± 4.17	
Invasive Ductal Carcinoma	226	8.58 ± 17.66		422	21.65 ± 45.35	
Invasive Lobular Carcinoma	12	4.88 ± 5.52		7	12.98 ± 28.98	
Mucinous Carcinoma	8	9.51 ± 8.87		12	8.98 ± 10.24	
**ER status**			0.120			0.525
Negative	74	9.29 ± 16.99		133	21.84 ± 50.18	
Positive	180	8.72 ± 19.75		338	18.08 ± 36.10	
**PR status**			0.485			0.632
Negative	119	9.42 ± 20.32		202	21.57 ± 48.21	
Positive	139	9.31 ± 17.50		199	14.25 ± 26.95	
**HER2 status**			0.123			0.019
Negative	178	9.58 ± 20.13		321	15.72 ± 31.92	
Positive	76	7.25 ± 15.87		132	29.09 ± 57.04	
**T**			0.909			0.492
T1	67	8.42 ± 16.36		163	16.9 ± 3.75	
T2	161	9.92 ± 21.22		254	22.62 ± 46.22	
T3/T4	23	3.71 ± 3.75		49	13.93 ± 25.36	
**N**			0.247			0.673
N0	119	8.39 ± 15.76		235	18.24 ± 38.50	
N1	75	11.28 ± 24.25		132	18.05 ± 36.92	
N2	30	6.65 ± 12.75		54	23.12 ± 50.08	
N3	28	6.96 ± 21.65		46	27.90 ± 54.21	
**Metastasis**			0.403			0.740
No	124	7.45 ± 19.48		277	18.57 ± 17.32	
Yes	10	2.46 ± 1.28		34	20.02 ± 51.03	
**Stage**			0.391			0.126
Stage 0	5	5.26 ± 2.83		2	3.44 ± 1.45	
Stage I	109	8.63 ± 13.69		212	18.89 ± 39.79	
Stage II	84	10.80 ± 23.01		140	17.67 ± 36.20	
Stage III/V	61	6.59 ± 17.03		117	23.25 ± 48.91	
**Grade**			0.360			0.320
Low	42	6.06 ± 10.59		97	15.88 ± 29.35	
Moderate	125	12.04 ± 36.07		221	16.74 ± 37.32	
High	80	8.44 ± 16.06		109	31.55 ± 58.78	
**Survival**			0.245			0.813
Alive	251	8.96 ± 19.05		441	18.85 ± 39.67	
Dead	11	3.29 ± 4.10		37	26.59 ± 8.79	
**Recurrence**			0.129			0.979
No	97	11.29 ± 39.20		216	18.80 ± 36.65	
Yes	10	6.67 ± 15.18		22	10.13 ± 12.92	

^#^Data were obtained from 766 BC patients divided into N > T and T > N groups

^§^Data are presented as the mean ± SE. One-way ANOVA was performed for statistical analysis of pathology, N status, stage, and grade data. Statistical analysis of other data was performed using the two-tailed Mann‒Whitney U test

HNMT protein levels in TMAs containing human breast tumor tissue (*n* = 211) were evaluated by IHC staining analysis (Additional file 2: Fig. S2A-C). The results of the IHC tissue scoring indicate a positive correlation between higher expression of HNMT protein and HER2 + BC patients. However, no correlation was observed with TNBC patients (Table 2 and Fig. 1F). In TMAs, HNMT protein expression levels were significantly higher in HER2 + (IHC score > 2 +) tumor tissues than in TNBC tumor tissues (Fig. 1G-H). Higher HNMT protein expression levels in the tumor tissues of BC patients were associated with poorer 10-year overall survival (Fig. 1I). The results from the TCGA database showed that high expression of *HNMT* mRNA in tumor tissues was associated with poorer prognosis in BC patients. Patients with high expression of both HNMT and HER2 (HR = 1.1, *P* = 0.59, *n* = 1070; Additional file 2: Fig. S2D-E) were especially prevalent. Previous studies have effectively utilized the non-competitive inhibitor of HNMT enzyme (Ki, 0.9 µM) SKF-91488 to block tissue histamine metabolism in experimental mice [39]. Therefore, we employed a high concentration (100 µM) of SKF-91488 to observe whether inhibiting HNMT enzyme activity affects the growth of cancer cells. We selected HER2 + BCLs (SKBR3 and BT474), TNBC (MDAMB-231), and normal breast epithelial cells (MCF-10A), and confirmed that the growth of all cell lines was unaffected under treatment with 100 µM SKF-91488 (Additional file 2: Fig. S2F). These results suggest that the overexpression of HNMT and HER2 is associated with poor clinical prognosis in BC patients.

**Table 2:** Correlations of the fold changes in HNMT protein expression with the clinical features of patients with BC

Parameter	n	Low expression (#)	High expression (#)	χ^2^	*P* value
		Mean (§) (n%)	Mean (§) (n%)		
**Age**				1.181	0.208
< 55 years	111	97.15 (53.2%)	234.53 (46.8%)		
≥ 55 years	86	106.75 ()45.3%	234.43 (54.7%)		
**ER status**				1.845	0.174
Negative	71	99.83 (43.7%)	245.40 (56.3%)		
Positive	115	102.34 (53.9%)	128.33 (46.1%)		
**PR status**				1.377	0.241
Negative	94	100.71 (45.7%)	239.54 (54.3%)		
Positive	92	102.14 (54.3%)	231.13 (45.7%)		
**HER2 status**				18.668	< 0.001
Negative	99	100.82 (59.6%)	207.65 (40.4%)		
Positive	41	119.58 (19.5%)	248.16 (80.5%)		
**TNBC Subtype**				0.075	0.784
No	34	122.67 (75%)	233.33 (25%)		
Yes	34	97.17 (93.5%)	200.00 (6.5%)		
**T**				2.568	0.463
T1	20	107.64 (60%)	244.26 (40.0%)		
T2	88	101.68 (53.4%)	236.38 (61.5%)		
T3	23	107.26 (39.1%)	231.00 (60.9%)		
T4	12	80.00 (41.7%)	233.33 (58.3%)		
**N**				2.560	0.464
N0	68	106.51 (52.9%)	243.88 (47.1%)		
N1	60	96.24 (48.3%)	235.89 (51.7%)		
N2	7	109.49 (71.4%)	200.00 (28.6%)		
N3	9	100.00 (33.3%)	218.57 (66.7%)		
**Metastasis**				4.002	0.120
No	140	105.77 (49.3%)	237.92 (50.7%)		
Yes	4	45.00 (100%)	0.00 (0.0%)		
**Stage**				0.660	0.719
Stage I	16	107.59 (56.2%)	253.96 (43.8%)		
Stage II	86	102.73 (51.2%)	237.52 (48.8%)		
Stage III/V	44	104.17 (45.5%)	233.05 (54.5%)		
**Survival**				3.347	0.048
Alive	130	104.56 (52.3%)	240.45 (47.7%)		
Dead	47	91.66 (36.4%)	227.11 (63.6%)		
**Recurrence**				1.230	0.267
No	116	107.31 (50.0%)	247.63 (50.0%)		
Yes	47	85.92 (40.4%)	220.00 (59.6%)		

^#^BC patients were divided into two groups according to their HNMT histo-scores in the TMA (low/below average vs. high/above average)

^§^Mean fold difference in HNMT protein expression in each group. The data are the means (n%). *P* values ​​for all the data were analyzed using Pearson's chi-square test

### HNMT overexpression enhances HER2-mediated tumor malignancy

**Fig. 2:**
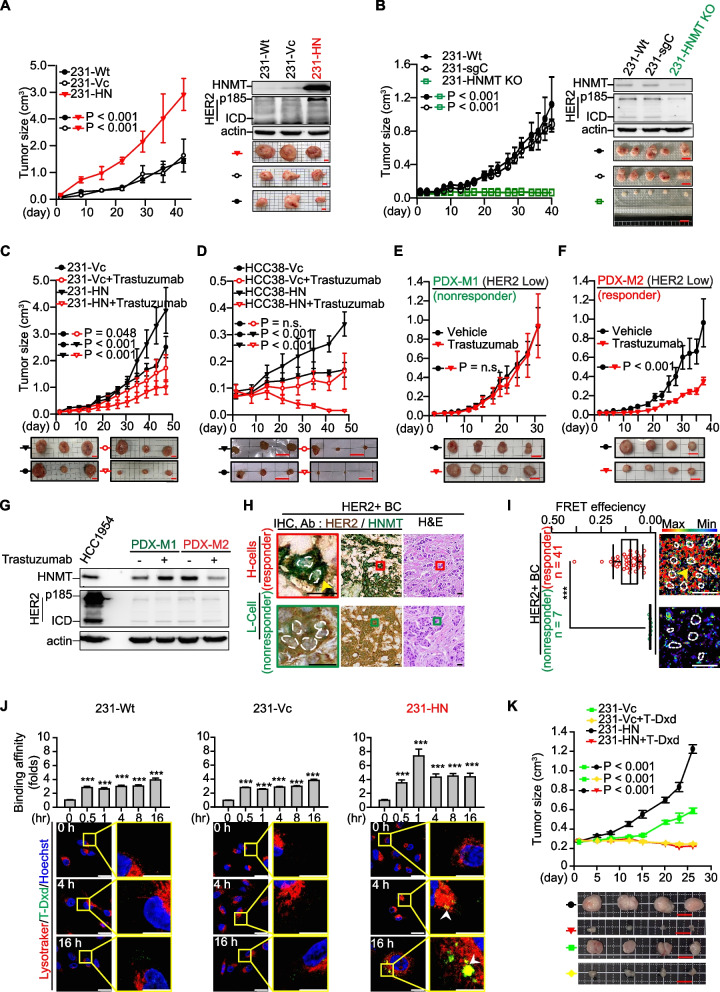
HNMT protein expression in trastuzumab- or T-Dxd-treated tumors. **A** The tumor growth curve of MDA-MB-231 xenograft tumors with HNMT overexpression; *n* = 3 per group. **B** Tumor growth curve after HNMT gene was knocked out in MDA-MB-231 (231-HNMT KO) transplanted tumors; *n* = 5 in each group. The protein levels of HNMT and HER2 in tumor tissues were detected by western blot. **C-F** Groups of HNMT-overexpressing TNBC cell xenograft tumors, including MDA-MB-231 (**C**) and HCC38 (**D**) tumors, *n* = 3 per group. Additionally, HER2-low PDX xenograft tumors are denoted as M1 (**E**) and M2 (**F**), *n* = 4 per group. The tumor groups underwent treatment with trastuzumab to evaluate the tumor growth curves. Trastuzumab was administered at 15 mg/kg twice weekly as part of the assessment process. **G** HNMT and HER2 protein expression in tumor tissues was detected by western blotting. **H** Representative images of H&E and HNMT/HER2 dual IHC staining of tumor tissue from HER2 + BC patients. **I** Representative FRET images (right) and quantitative results of FRET efficiency (left) in HER2 + tumor sections. **J** After treatment with 10 μg/mL T-Dxd, the cells were subjected to flow cytometry analysis at various time points (0, 0.5, 1, 4, 8, 16 h, as shown in the upper panel) and IF staining at different time points (0, 4, 16 h, as shown in the lower panel). The flow cytometry analysis revealed that the duration of treatment influenced the binding affinity of T-Dxd to the cells. T-Dxd binding to the cells was visualized as green fluorescence in the IF staining experiment. At the same time, LysoTracker exhibited red fluorescence, and Hoechst displayed blue fluorescence, enabling the localization of T-Dxd within the cells. These experimental results will contribute to assessing T-Dxd's binding affinity for BC. The experiments were conducted independently three times. **K** The TNBC cell-derived xenograft tumors were randomly assigned to either the untreated group or the group receiving anti-HER2 therapy (T-Dxd, 4 mg/kg, administered once weekly, *n* = 4 per group). Tumor growth curves were provided for both groups. The data are the means ± SE. Statistical analysis was performed using a two-tailed unpaired Student's t-test. ***P < 0.001. Scale bars = 0.8 cm (**A, C**), 1 cm (**B**, **E**, **F**, **K**), 1.13 cm (**D**), 10 µm (**H**), 20 µm (**I**), and 18.4 µm (**J**)

We next asked whether HNMT overexpression affects HER-2-mediated tumor aggressiveness. We selected TNBC cells as the research model (Fig. 1A, *n* = 9). Among these TNBC cells, MDA-MB-231 cells expressed relatively high levels of HNMT protein and low levels of HER2. Indeed, we observed that forced overexpression of HNMT accelerated the growth of xenograft tumors derived from MDA-MB-231 cells (referred to as 231-HN), in which the HER2 protein was increased in the tumor tissues (Fig. 2A). On the other hand, we have successfully utilized CRISPR-Cas9 technology to establish an HNMT gene knockout model in MDA-MB-231 (named 231-HNMT KO) BC cells. Based on our observations, we have found that the deletion of the HNMT gene significantly inhibits the growth rate of tumors compared to the control group (231-sgC) (Fig. 2B). The malignant phenotype of these cells also changed according to HNMT status (Additional file 3: Fig. S3A-B). Interestingly, we found that overexpression of HNMT promoted lung metastasis in established primary cultured 231-HN-Luc cells (yellow arrow, Additional file 3: Fig. S3C). The results showed that transplantation of 231-HN cells into NSG mice resulted in more lung metastatic nodules than in 231-Vc tumor-bearing mice (Additional file 3: Fig. S3C). These results suggest that HNMT overexpression increases the aggressiveness of BCL.

According to our analysis of cancer tissues from xenograft mice, the overexpression of HNMT in 231-HN cells resulted in a significant increase in HER2 expression compared to the control group (231-Vc) cells (Fig. 2A). Further cell experiments confirmed the association between HNMT overexpression and the malignancy of cancer cell metastasis. However, in these HNMT-overexpressing cells, the expression of EGFR (HER1) and HER3 proteins did not change (Additional file 3: Fig. S3B). To confirm that the invasiveness of lung metastasis induced by HNMT expression is related to HER2 overexpression, we established primary cultured cells from mouse lung metastasis tissues with 231-HN-derived xenograft tumors, named 231-HN-LM cells. The same results were also observed in primary cultured cells isolated from 231-HN-derived xenograft (231-HN-XE) and lung metastasis (231-HN-LM) cells (Additional file 3: Fig. S3D). We confirmed these observations by inhibiting HNMT protein expression in 231-HN cells via siRNA (231-HN-Si). As a result, we found decreased levels of HER2 and its regulatory proteins, such as p-AKT (Additional file 3: Fig. S3E). Interestingly, we observed a significant increase in T-AKT in 231-HN cells. We conducted RNA sequencing analysis to understand the mechanism further and collected data from 231-Vc and 231-HN cells. The results showed that in 231-HN cells, various proteins associated with cell growth, including E2F1, AP1, STAT3, and GLI1, were upregulated (Additional file 3: Fig. S3F). Previous studies have demonstrated that E2F1 transcription can upregulate T-AKT protein expression [40]. To confirm whether the upregulation of E2F1 genes induced by HNMT overexpression is involved in T-AKT protein expression, we further treated 231-HN cells with E2F1-specific inhibitor (HLM006474). We confirmed a reduction in T-AKT expression in these cells after treatment with the E2F1 inhibitors (Additional file 3: Fig. S3F). These results indicate the involvement of E2F1 in T-AKT expression in 231-HN cells. Therefore, the above results support the idea that increased HER2 expression in 231-HN tumor cells due to HNMT overexpression may be associated with increased sensitivity to trastuzumab. Based on the above results (Additional file 3: Fig. S3F), we hypothesized that AKT plays a vital role in the HNMT/HER2 oncogenic message. Therefore, we treated cells overexpressing HNMT (231-HN) and control cells (231-Vc) with EGF (100 ng/mL) for 24 h and then performed RNA-Seq analysis. According to expectations [41], this data is consistent with previously published research results, demonstrating similar comparative outcomes for activating the PI3K/Akt or MAPK pathways in HER2 + tumors with the PEDERSEN gene. (NES = 1.24, *P* value = 0.04). The GSEA data was also presented (Additional file 3: Fig. S3G).

### HNMT overexpression in cancer cells increases sensitivity to trastuzumab therapy

Based on the observations in Figs. 1 and 2A, we propose that the increased expression of HNMT in BC cells corresponds to the increased expression of HER2. In our study, we successfully generated stable SKBR3 cell lines with varying levels of HNMT, including HNMT-overexpressing, HNMT-Si, and HER2-Si cells. Our findings revealed that cells overexpressing HNMT exhibited increased expression of HER2 protein, whereas cells with depleted HNMT showed reduced expression of HER2 protein. In contrast, the HNMT protein level showed no difference between HER2-Sc- and HER2-depleted cells (Additional file 4: Fig. S4A). To this end, we established a trastuzumab-responsive MDA-MB-231 cell line (231-DOX-HN) by overexpressing the HNMT protein using a Tet-on system (Additional file 4: Fig. S4B-C). HNMT overexpression increased trastuzumab binding in DOX-induced 231-DOX-HN cells (Additional file 4: Fig. S4D). Furthermore, stable HNMT-overexpressing BC cells (HN cells) were established using the pcDNA5/TO system. pcDNA5/TO was used to select stable cells as vector control (Vc) cells. HER2 expression levels were increased in HNMT-overexpressing TNBC cells (Additional file 4: Fig. S4E). Trastuzumab binding increased when HNMT was overexpressed in various TNBC (HN) cell lines (Additional file 4: Fig. S4F). These results support our hypothesis that HNMT proteins can alter HER2 protein expression, which may, in turn, affect the response of BC cells to trastuzumab treatment. Modulating the expression of HNMT provides us with a new strategy to enhance the sensitivity of BC cells to trastuzumab treatment.

To further validate whether HNMT expression can enhance sensitivity to trastuzumab treatment in vivo, we selected two HNMT-overexpressing TNBC cell lines (231-HN and HCC38-HN) to establish a trastuzumab-responsive xenograft tumor model. The results showed that established TNBC tumors with high HNMT expression were more sensitive to trastuzumab treatment than were those in the vector control group (Fig. 2C-D). To validate the results of this study, we purchased two tumor xenograft mice derived from patients' tumor tissues (PDXs) with low HER2 expression (IHC score 2 + , *n* = 8 each). Model 1 (PDX-M1) and Model 2 (PDX-M2) were named to test their response to trastuzumab treatment. We found that the HER2 and HNMT protein levels were higher in the tumor tissues of trastuzumab-treated responders (PDX-M2) than in those of non-responders (PDX-M1) (Fig. 2E-G).

Based on these data, we hypothesized that both HER2 and HNMT are overexpressed in the tumor tissue of patients who respond to trastuzumab. This hypothesis was validated by observing membrane-localized HER2 and cytoplasmic-localized HNMT in the same individual tumor cell lines via IHC double-staining. We selected tumor tissues from BC patients treated with trastuzumab and classified them into responders and non-responders (n = 3 per group, Additional file 5: Fig. S5A). Tumor tissue was immunochemically stained to observe the protein expression of HNMT and HER2 in tumor cells. Higher levels of cytoplasmic HNMT (detected by HNMT-specific antibody, stained green) and membranous HER2 (detected by trastuzumab, stained brown) proteins were detected in the cancer cells of all responder tumor sections. Therefore, we defined these IHC staining phenotypes as H-cells (Fig. 2H, upper panel, and Additional file 5: Fig. S5A, left panel, yellow arrows). In contrast, cells with lower cytosolic HNMT and membrane-localized HER2 were named L-cells (Fig. 2H, bottom panel, and Additional file 5: Fig. S5A, right panel). Similar results were observed in tumor tissues from HER2-low PDX (PDX-M1, PDX-M2) and TNBC xenograft tumors (Additional file 5: Fig. S5B-C). The results showed that the H-cell phenotype expressed in tumor tissues was associated with a favorable response to trastuzumab treatment (Additional file 5: Fig. S5, left panel).

### HER2 and HNMT complex formation for the identification of trastuzumab treatment responders

Our previous studies showed that the HER2 protein participates in the carcinogenesis of BC through membrane protein interactions and determines the prognosis of patients [34, 35]. The interaction of HER2 with increased HNMT protein expression in H-cells could be a promising indicator for identifying trastuzumab responders. We mined three independently published microarrays to discover associations between HNMT expression and patient response to trastuzumab treatment [42]. Patients with HER2 + BC who received trastuzumab-based therapy in three cohorts had a pathological complete response (pCR), defined by their clinical outcomes as the absence of invasive cancer or residual disease (RD) in the breast and axilla. Interestingly, the pCR patients had higher *HNMT* mRNA expression levels than the RD patients (Additional file 6: Fig. S6A). To validate this observation, we retrospectively collected tumor tissue from HER2 + BC patients treated with trastuzumab. We analyzed the formation of HER2 and HNMT complexes in tumor tissues by FRET experiments. Trastuzumab treatment responders (*n* = 41) had significantly higher FRET efficiencies than non-responders (*n* = 7) (Fig. 2I, and Additional file 6: Fig. S6B-C indicated by yellow arrows). This result suggested that the formation of HER2/HNMT complexes in tumor sections is a promising predictor of the response to trastuzumab therapy in BC patients.

### HNMT overexpression in cancer cells increases sensitivity to therapy with antibody‒drug conjugates (ADCs) targeting HER2

According to a previous paper [43], a phase 3 clinical trial in a group of patients with HER2-low metastatic BC patients with TNBC responded to T-Dxd. The T-Dxd-treated TNBC patients had significantly longer progression-free survival (PFS) and overall survival (OS) than patients in the physician's chemotherapy group. However, the underlying mechanism remains unclear, and this study inspired us to confirm whether HNMT expression in TNBC tumor tissue can be used as a molecular marker to identify patients who respond to anti-HER2 ACD (T-Dxd) therapy. We analyzed the HNMT protein level in several TNBC cell lines (Fig. 1A, *n* = 9). Among these TNBC cells, only MDA-MB-231 cells expressed relatively high levels of the HNMT protein (Fig. 1A). MDA-MB-231 cells (231-Wt, 231-Vc, and 231-HN) were treated with an anti-HER2 ADC (T-Dxd) [42] to determine the IC_50_ values. The in vitro IC_50_ results showed that 231-HN cells were more sensitive to T-Dxd treatment than 231-Vc cells but far less sensitive than HER2 + (HCC1954) cells (Additional file 7: Fig. S7A). IF staining and flow cytometry were used to analyze whether the H-cell phenotype is related to T-Dxd drug uptake. The results showed that 231-HN and HCC1954 cells took up T-Dxd faster than 231-Wt cells, internalized T-Dxd into endosomes, and accumulated in lysosomes after 4 to 16 h (Fig. 2J and Additional file 7: Fig. S7B, white arrow). According to the above results (Additional file 3: Fig. S3D), the expression of the HER2 protein was also increased in TNBC cells (231-HN) overexpressing HNMT. We believe that 231-HN cells can increase the binding of T-Dxd to 231-HN cells by increasing the level of the HER2 protein. Based on these results, we hypothesized that the H-cell phenotype could be used to select a subset of TNBC patients for anti-HER2 therapy.

We established 231-Vc and 231-HN cell xenograft tumor models to test the response to T-Dxd treatment. The results showed increased sensitivity of 231-HN tumors to T-Dxd compared to untreated 231-HN tumors (Fig. 2K, red vs. black lines). Surprisingly, we found that T-Dxd treatment inhibited the growth of 231-Vc-derived xenograft tumors (Fig. 2K, green vs. yellow lines). From the results shown in Fig. 1A, we speculate that although the expression of the HNMT protein in 231-Vc cells is lower than that in HER2 + BC cells, these cells can still exhibit a minor H-cell phenotype. To explore the correlation between the H-cell phenotype of TNBC tumors and their sensitivity to the therapeutic effect of T-Dxd, tumor tissues were dissected at the end of the experiment, and IHC staining was performed. Unexpectedly, 231-Vc-derived xenograft tumors in the T-Dxd-treated group had increased HNMT protein levels in cells exhibiting a more prominent H-cell phenotype compared with the untreated group (*n* = 4, Additional file 7: Fig. S7C, red vs black bar). According to this study's findings, the expression of HNMT within tumor cells is activated during T-Dxd treatment.

The results indicate that HNMT may be a critical factor in determining the effectiveness of T-Dxd treatment. To confirm whether T-Dxd treatment of TNBC tumors can increase the amount of the HNMT protein in cells. We selected MDA-MB-231 cells and treated them with T-Dxd (10 μg/mL) for 6 days. Western blot analysis confirmed increasdn HNMT expression in MDA-MB-231 cells, indicative of the H-cell phenotype (Additional file 7: Fig. S7D). To further test whether HNMT expression in MDA-MB-231 xenograft tumor tissues increases T-Dxd binding to tumor cells in vivo, we obtained T-Dxd-treated xenograft tumor tissues and performed ex vivo IHC staining with anti-T-Dxd antibodies. The results showed that the binding of 231-Vc- and 231-HN-derived tumor tissue to T-Dxd increased significantly after T-Dxd treatment (Additional file 7: Fig. S7E, yellow arrow). The above results indicate that T-Dxd treatment is valuable for the small number of patients with TNBC tumors that express the H-cell phenotype.

### Epidermal growth factor (EGF) receptor-mediated complex formation of HER2 and HNMT involved early-step carcinogenesis

**Fig. 3:**
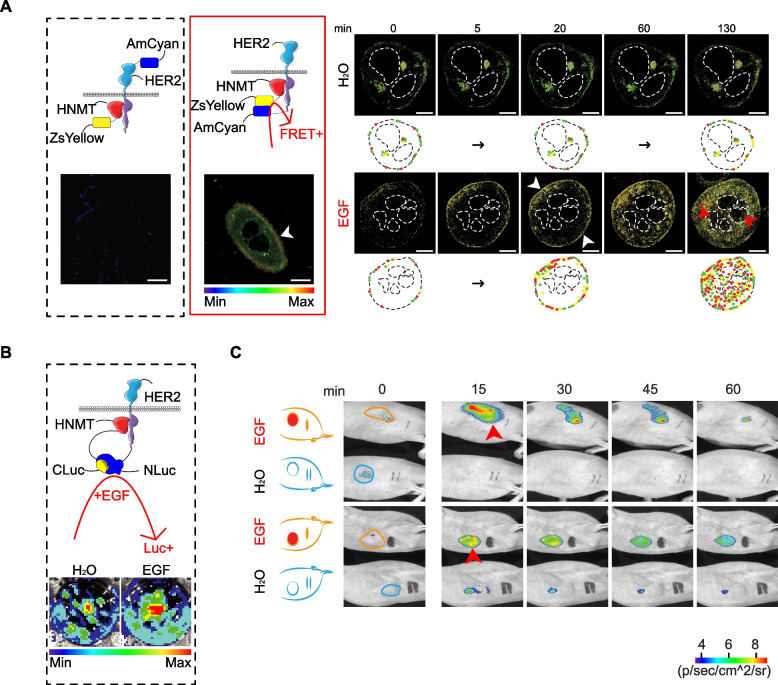
Time-dependent dynamics of the HNMT and HER2 interaction in BC cells. **A** Time-lapse FLIM-FRET images (0, 5, 20, 60, 130 min) of HNMT and HER2 complex formation in control and EGF-treated cells. We labeled the blue fluorescent protein (AmCyan) at the N-terminal (AmCyan-HER2) and C-terminal (HER2-AmCyan) of the HER2 protein. In contrast, the C-terminal of the HNMT protein was labeled with a yellow fluorescent protein (ZsYellow). We used different combinations of HER2 protein fragments and HNMT to observe the formation of HNMT/HER2 protein complexes. Black box: AmCyan-HER2 and HNMT-ZsYellow; red box: HER2-AmCyan and HNMT-ZsYellow. Scale bar = 7.5 µm. **B** The HER2 protein was labeled with N-terminally cleaved luc protein (N-Luc), while the HNMT protein was labeled with C-terminally cleaved luc protein (C-Luc). Luc activity was detected using bioluminescence images (bottom) after 30 min of treatment with 100 ng/mL EGF. **C** Time-lapse bioluminescence images (0, 15, 30, 45, 60 min) in vivo of mice bearing luc-carrying cancer cells (Fig. 3B) after treatment with EGF. *n* = 2 per group

Elucidating the mechanism by which the upregulation of HNMT induces BC formation is important for future drug development. First, it was demonstrated in a previous paper that EGF treatment stimulated HER1-mediated translocation of HNMT from the cytoplasm to the cell membrane [30]. After treating SKBR3 cells with 100 ng/mL of EGF, we observed the translocation of HNMT to the cell membrane within approximately 20 min using the Western blotting technique (Additional file 8: Fig. S8A). We further utilized the IP technique to assess whether EGF treatment induces protein complex formation between HNMT and HER1 or HER2 at 20 min post-treatment. Our results demonstrate that HNMT forms protein complexes with HER2 following EGF treatment (Additional file 8: Fig. S8B). In the FRET analysis (Additional file 8: Fig. S8C, yellow arrows), the formation of HER2 and HNMT complexes at the plasma membrane induced by EGF (or NRG1) was detected within 20 min. A live-cell FLIM assay was performed to verify the intracellular localization of HER2 and HNMT complex formation time-dependent. In this study, the HER2 protein was labeled with a blue fluorescent protein (AmCyan) either at the N-terminus (Fig. 3A, black box) or C-terminus (Fig. 3A, red box). The HNMT protein was labeled with a yellow fluorescent protein (ZsYellow) at the C-terminus. The results indicated that the HNMT and HER2 fluorescent proteins interact tightly in the inner membrane of SKBR3 cells as early as 5–20 min after treatment with EGF (Fig. 3A, white arrow, and Additional file 8: Fig. S8D), as evidenced by a marked increase in the FRET signal. In this experiment, we repeated the experiment and used FRET technology to confirm that HNMT formed a weak interaction with HER1 after EGF treatment (Additional file 8: Fig. S8E). After knocking out the HER1 gene and inhibiting the dimerization of HER1/HER2, we observed a significant inhibition of the interaction between HNMT and HER2 through FRET analysis (Additional file 8: Fig. S8F). Based on the above results, it is evident that EGE can induce the formation of HER1/HER2 dimers, whereas HNMT exhibits a greater propensity to interact with HER2 only under this condition. The HER2 and HNMT complexes then translocated into the cytoplasm 60–130 min after EGF exposure (Fig. 3A).

Split-luc experiments were performed to validate the formation of HER2 and HNMT complexes in vivo using the method described in our previous paper [35]. We split luc into two fragments that attach to the C-termini of the HNMT and HER2 proteins. EGF-induced HNMT/HER2 complex formation was detected based on luc activity (Fig. 3B). Using the SKBR3 cells stably expressing luc described above (Fig. 3B), we established an in vivo mouse xenograft model (Fig. 3C). Tumor-bearing mice were divided into two groups (Fig. 3C, *n* = 2 per group). We treated two mice with EGF and detected significant luc activity at tumor sites (red arrows) in both groups at 15 min. However, control mice treated with H_2_O had no detectable luc activity after 60 min (Fig. 3C). This result suggested that an early step in HER1-mediated complex formation of HER2 and HNMT occurs in tumor tissue.

### HNMT promotes cytosolic HER2-ICD shedding in EGF-treated SKBR3 cells

**Fig. 4:**
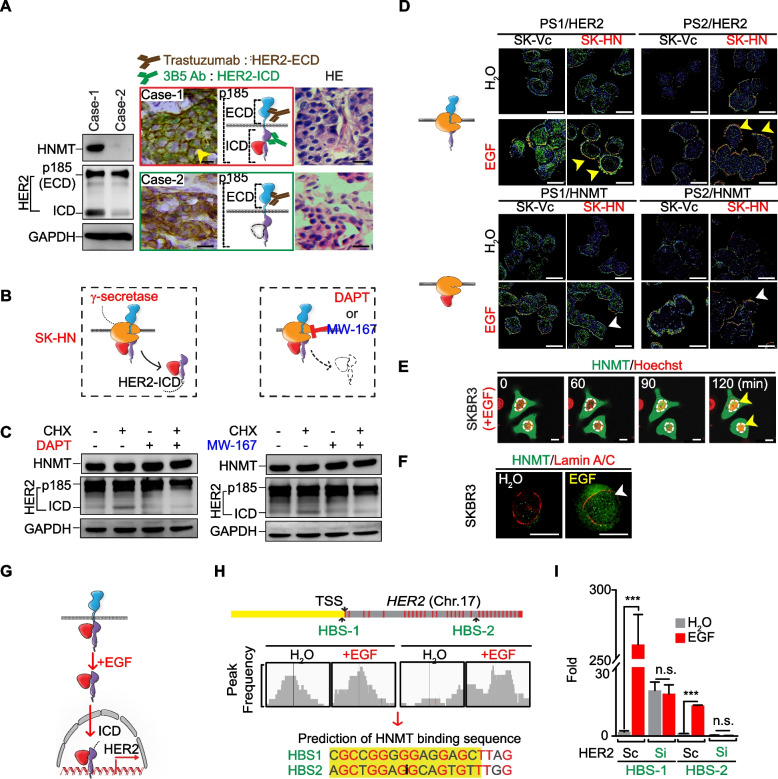
Cytoplasmic HER2-ICD shedding and nuclear translocation in H-cells. **A** Endogenous expression of the HNMT and HER2 proteins in two HER2 + BC tumor tissues was detected by western blot analysis (left) and IHC double staining (right). Cytosolic HER2-ICD (stained green, yellow arrows) and membranous HER2-ECD (stained brown) were detected by specific antibodies as indicated. H&E staining of BC tumors is shown. **B** Schematic illustration of the inhibition of HER2-ICD shedding by γ-secretase inhibitors (DAPT and MW-167). **C** Western blot confirming the expression of indicator proteins with/without CHX or γ-secretase inhibitor (10 μM, 24 h) in SKBR3 cells overexpressing HNMT. **D** Representative FRET images of SK-HN and SK-Vc cells treated with/without EGF (100 ng/mL, 60 min) confirm that HER2 (top) or HNMT (bottom) interacts with PS1 or PS2. The yellow (for HER2 and the PS1 or PS2 complex) and white (for HNMT and the PS1 or PS2 complex) arrows indicate a positive FRET signal. **E** Time-lapse fluorescence images (0, 60, 90, 120 min) of HNMT in SKBR3 cells expressing HNMT-ZsYellow treated with 100 ng/mL EGF. Yellow arrows indicate nuclear translocations. **F** Fluorescence images consisting of 35 x–y frames were captured every 0.5 μm on the y-axis using a deconvolution microscope. Representative fluorescence images of SKBR3 cells treated with and without 100 ng/mL EGF (120 min). The white arrows indicate nuclear translocation. **G** Schematic representation of EGF-induced HAP activation of the *HER2* promoter. **H** Cells were treated with 100 ng/mL EGF for 120 min. Schematic representation of the distribution of HBS-1 and HBS-2 in the *HER2* gene (top). Snapshot of IGV showing the frequency of detection by ChIP-sequencing analysis of the HBS-1 and HBS-2 loci (middle). Potential sequences of HBS-1 and HBS-2 are shown after the alignment of ChIP-sequencing data (bottom). **I** ChIP real-time PCR analysis of HER2-Sc and HER2-Si cells with or without EGF treatment. The data are presented as the mean ± SE. Statistical analysis was performed using a two-tailed unpaired Student's t-test. ***P < 0.001. Scale bars = 20 μm (**A**), 10 µm (**D, E**), and 10 μm (**F**)

It has been previously shown that HER2-mediated downstream oncogenic signaling in BC cells is activated by nuclear translocation of the truncated receptor tyrosine kinase (RTK) p95 intracellular soluble domain (i.e., p95HER2, named HER2-ICD) [44, 45]. Therefore, we hypothesized that extracellular oncogenic signaling drives HNMT to initiate HER2 protein shedding (HER2-ICD formation). To evaluate this hypothesis, two BC tumor sections were randomly chosen based on having an equivalent level of HER2 positivity but varying levels of HNMT protein (Fig. 4A, left panel). The full-length HER2 protein (p185HER2) is primarily distributed on the cell membrane, while its truncated form HER2-ICD (p95HER2) is mainly distributed in the cytoplasm or nucleus. Then, the intracellular localization of HER2 in these two tumor sections was examined by dual IHC staining using different antibodies. The anti-HER2 (3B5) antibody demonstrates specific recognition of the HER2 protein at the 1242–1255 amino acid region, forming the HER2-ICD fragment and presenting as green staining. In contrast, the trastuzumab monoclonal antibody can target the extracellular domain of HER2 and present as brown staining (Fig. 4A, right panel). Based on the analysis of breast tumor tissue, it is evident that in the case of high expression of HNMT protein (case 1), HER2-ICD was detected in the cytoplasm (stained in green) along with an equivalent amount of HER2 extracellular domain (HER2-ECD, stained in brown). Conversely, in breast tumor tissue lacking HNMT protein expression (case 2), a reduction in the formation of HER2-ICD was observed, with only the extracellular HER2-ECD region being detectable (stained in brown) (Fig. 4A, middle panel). These results suggest that HNMT proteins promote cytosolic HER2-ICD shedding in HER2 + BC cells.

As described above, we found that EGF treatment of BC cells rapidly drove the interaction of cytoplasmic HNMT with HER2 on the inner membrane within 15–20 min (Fig. 3A). Our hypothesis posits that the interaction between HNMT and HER2 leads to the recruitment of a specific protease, γ-secretase, which in turn cleaves the amino acid K676 within the nuclear localization signal (NLS) [46] fragment located near the juxtamembrane region of the HER2 protein (Fig. 4B and Additional file 9: Fig. S9A) to form the carboxy-terminal fragment (HER2-ICD) (Additional file 9: Fig. S9B, left) [41]. This hypothesis could explain the presence of HER2-ICD in the cytoplasm of tumor tissues overexpressing HNMT (Fig. 4A, Patient 1). Similarly, HER2-ICD was exclusively detected in HNMT-overexpressing (SK-HN) cells, while it was not detected in HNMT-inhibited (SK-Si) cells subjected to CHX treatment (Additional file 9: Fig. S9B, right). CHX precludes the involvement of newly synthesized proteases in the cytoplasmic shedding of HER2-ICD mediated by γ-secretase. Notably, the administration of γ-secretase inhibitors (specifically DAPT and MW-167) in conjunction with CHX to SKBR3 cells resulted in a notable reduction in the shedding of HER2-ICD (Fig. 4C).

These findings indicate that γ-secretase plays a role in releasing HER2-ICD in HNMT-overexpressing cells through protein–protein interactions. The results of IP experiments provided further evidence of the interaction between γ-secretase and HNMT in SKBR3 cells (Additional file 9: Fig. S9C). Upon EGF treatment, a noticeable elevation in the interaction between HNMT and γ-secretase components, particularly presenilin 1 (PS1) and presenilin 2 (PS2), was observed in SK-HN cells via FRET analysis (Fig. 4D). The activation of HER2-ICD and the formation of HER2-p185 in SK-HN cells are promoted by EGF, as shown in Additional file 9:Fig. S9D. However, the expression of HER2-ICD is significantly reduced regardless of the presence of EGF when γ-secretase is inhibited (SK-HN/PS1/2-Si), as demonstrated in Additional file 9: Fig. S9D and S9E. These findings strongly indicate the crucial role of γ-secretase in regulating HNMT-mediated HER2-ICD expression. Our investigation revealed a notable increase in the interaction between HNMT/HER2, HNMT/PS1/2, and HER2/PS1/2 following EGF treatment in SK-HN cells, as evidenced by FRET analysis (Fig. 4D). Upon examination of the findings, it is apparent that the exposure of BC cells to EGF leads to the cytoplasmic binding of HNMT to HER2, subsequently facilitating the recruitment of γ-secretase components (PS1 or PS2) for the generation of HER2-ICD within the cytoplasm.

### HNMT promotes HER2-ICD nuclear translocation in EGF-treated SKBR3 cells

Previous research has demonstrated that activating cell survival signals via EGF leads to the translocation of the HER2-ICD protein from the cell membrane to the nucleus [44, 47]. Based on these findings, we formulated a potential mechanism through which EGF can stimulate HNMT to facilitate the nuclear translocation of the HER2-ICD protein. Live-cell confocal microscopy was performed to visualize the HNMT protein labeled with a fluorescent protein (ZsYellow). Figure 4E shows the notable nuclear translocation of the HNMT-ZsYellow fluorescent fusion protein in SKBR3 cells following 120 min of treatment with EGF, as indicated by the yellow arrowheads. Furthermore, IF staining was conducted using a DeltaVision Elite Deconvolution microscope to observe the EGF-induced nuclear translocation of the HNMT protein, as depicted by the white arrow in Fig. 4F. We performed IF double staining experiments using HNMT and HER2-ICD specific (3B5) antibodies and conducted quantitative colocalization analysis. The results showed that HNMT and HER2-ICD colocalization was evident in the nuclei of EGF-treated SK-HN cells. (Additional file 10: Fig. S10A, white arrow). Conversely, in the same experiment, IF double staining with HNMT and p185HER2-specific (trastuzumab) antibodies revealed no colocalization of HNMT and p185HER2 in the nucleus (Additional file 10: Fig. S10A, yellow arrows).

To investigate the nuclear translocation of the HNMT/HER2-ICD complex following EGF treatment and its role in the transcriptional activation of HER2 (Fig. 4G), we used HNMT-specific antibodies to immunoprecipitate HNMT-associated proteins (HAPs) from EGF-treated and untreated SKBR3 cells. Subsequently, we conducted ChIP sequencing to analyze the DNA sequences associated with HAPs. This approach allowed us to assess the potential involvement of the HNMT/HER2-ICD complex in modulating gene transcription, particularly that of HER2. The ChIP-sequencing data presented in Table 3 and Additional file 10 (Fig. S10B) revealed that the *HER2* promoter was the second most abundant gene fragment identified by HAP. Subsequently, two HAP binding sites, designated HBS-1 and HBS-2, were predicted within the *HER2* promoter, as illustrated in Fig. 4H. The binding site of HAPs on the *HER2* promoter was confirmed through ChIP‒PCR experiments conducted in EGF-treated SKBR3 cells using primers specifically designed from the *HER2* promoter sequence. The findings revealed that HAPs predominantly bind to the HBS-1 site on the *HER2* promoter (Fig. 4I). Additionally, in HER2-Si SKBR3 cells with reduced HER2 expression, the binding of HAPs to HBS-1 was notably diminished, thus validating the significance of HER2 as a critical component of HAPs involved in the transcriptional regulation of *HER2* gene expression (Fig. 4I).

**Table 3:** List of the top 50 target genes determined by ChIP sequencing

No	Strand	Transcript ID	Symbol	Reference (Coexpression with HER2)
1	-	NM_001257408	*IKZF3*	*Clin Transl Oncol* 2020, 22(4):576–584
2	+	NM_001005862	*ERBB2*	
3	+	NM_005310	*GRB7*	*Nature* 2000, 406:747–752 *J Clin Oncol* 2020, 38(5):462–471 *Ann Oncol* 2020, 31(suppl_4): S1052-S1064
4	-	NM_001291726	*PGAP3*	*Breast Cancer Res Treat* 2018, 172(2):313–326 *Ann Oncol* 2020, 31(suppl_4): S1052-S1064
5	+	NR_003594.7	*REXO1L2P*	
6	+	NM_032192	*PPP1R1B*	*Cancer Res* 2012, 72(17):4504–1 *Clin Cancer Res* 2018, 24(5):1216–1226.4
7	-	NM_000723	*CACNB1*	*Front Pharmacol* 2018, 3;9:861
8	-	NM_001025253	*TPD52*	*Mol Carcinog* 2014, 53(10):807–19
9	-	NM_003600	*AURKA*	*BMC Cancer* 2012, 12:562 *JAMA Oncol* 2017, 3(5):666–671
10	+	NM_001283024	*WDYHV1*	
11	+	NM_0007591	*CSF3*	
12	+	NM_001283012	*DEPTOR*	
13	-	NM_000791	*DHFR*	
14	+	NM_001278645	*NDUFB9*	
15	-	NM_000318	*PEX2*	
16	+	NM_001011720	*XKR9*	
17	-	NM_001017926	*ZHX1*	
18	+	NM_001033521	*CSTF1*	
19	-	NM_001002796	*MCTP1*	
20	-	NM_020405	*PLXDC1*	
21	+	NR_003367	*PVT1*	
22	+	NM_001165937	*STARD3*	*Nature* 2000, 406:747–752 *Ann Oncol* 2020, 31(suppl_4): S1052-S1064
23	-	NM_001146160	*TATDN1*	*Oncotarget* 2016, 7(14):18,219–28
24	+	NM_001277145	*ZBTB10*	
25	+	NM_015083	*CDK12*	*Nature* 2016, 534(7605):55–62 *Nucleic Acids Res* 2017,45(11):6698–6716
26	-	NM_001134671	*DERL1*	
27	+	NM_032899	*FAM83A*	*PLoS One* 2017, 12(5):e0176778
28	-	NM_001184906	*FBXL20*	
29	-	NM_001242463	*FBXO32*	
30	+	NM_052886	*MAL2*	
31	+	NR_039881	*MIR4728*	*Cancer Biomark* 2015, 15:807–814 *Mol Genet Genomics* 2019, 294(1):95–110
32	-	NM_138463	*TLCD1*	
33	-	NM_001308225	*ZNF521*	
34	+	NM_198844	*ZPBP2*	
35	-	NM_003657	*BCAS1*	
36	+	NM_001254	*CDC6*	*Endocr Relat Cancer* 2006, 13(1):39–49
37	+	NM_001359	*DECR1*	
38	-	NM_032339	*MIEN1*	*Oncotarget* 2017, 8(54):92,209–92226
39	-	NR_039913	*MIR4756*	
40	-	NR_031608	*MIR663B*	
41	-	NR_107045	*MIR8078*	
42	+	NM_002439	*MSH3*	
43	-	NM_001190470	*MTRNR2L2*	
44	-	NM_006540	*NCOA2*	
45	+	NM_178170	*NEK8*	
46	-	NM_139280	*ORMDL3*	
47	-	NM_173825	*RABL3*	
48	+	NM_000981	*RPL19*	*Cancer Res* 2003, 63(9):2194–9 *Oncol Rep* 2012, 28(1):365–9
49	-	NM_198993	*STAC2*	
50	+	NR_033770	*ROCK1P1*	

The results showed the 50 most frequently analyzed HAP binding sites and their corresponding target genes by ChIP sequencing

### The HNMT/HER2-ICD complex binds to the *HER2* promoter to enhance HER2 protein upregulation

A luc reporter assay was conducted to investigate the potential enhancement of HNMT-mediated upregulation of the HER2 protein through the binding of HNMT/HER2-ICD to the *HER2* promoter. For this purpose, plasmids 4X-HBS-1 and 4X-HBS-2 were specifically constructed for transfection into BC cells exhibiting different levels of HER2 protein expression, namely, HER2-Sc and HER2-Si (Additional file 10: Fig. S10C). In EGF- and NRG1-treated HER2-Sc cells, luc activity was significantly detected in cells transfected with 4X-HBS-1 but not 4X-HBS-2 plasmids. In contrast, no increase in luc activity was detected in HER2-Si cells transfected with 4X-HBS-1 or 4X-HBS-2 following EGF or NRG1 treatment. These findings suggest that HNMT/HER2-ICD complex nuclear translocation and binding to the HBS-1 site of the *HER2* promoter activate the transcriptional upregulation of the HER2 protein.

To test this hypothesis, a variety of HER2 + BCLs, such as SKBR3, AU565, BT-474, and HCC1954, were selected for overexpressing (HN) or suppressing (Si) HNMT protein expression, as depicted in Additional file 10 (Fig. S10D-E). Paired HER2 + BCLs with different HNMT protein expression levels were established to replicate the observed H- and L-cell phenotypes in tumor tissues, as shown in Additional file 10 (Fig. S10D). Upon analysis of established H- and L-cells using flow cytometry, the sensitivity of HER2 + BC cells to trastuzumab binding was examined. The results consistently correlated with the trastuzumab binding affinity and HNMT expression levels in all HER2 + BCLs (Additional file 10: Fig. S10E).

**Fig. 5:**
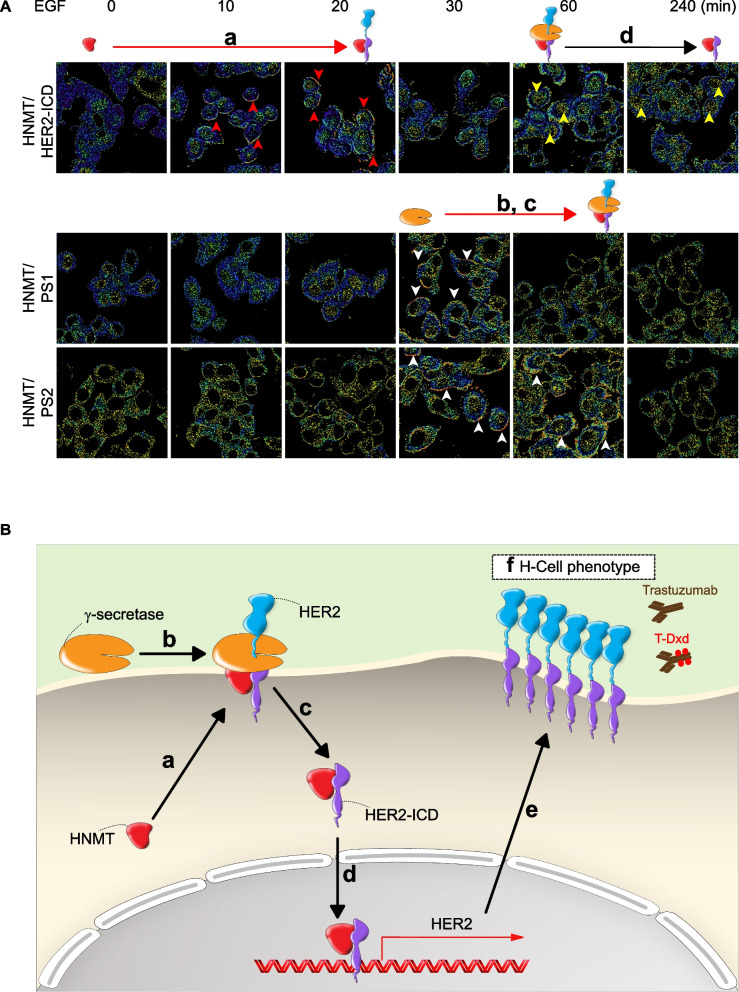
The molecular mechanism by which HNMT enhances BC cell sensitivity to anti-HER2 targeted drugs. The molecular mechanism underlying HER2-induced oncogenesis involves a series of intricate steps. **A** In this study, EGF (100 ng/mL) was added to SKBR3 cancer cells, and the kinetic process of complex formation was observed. (**a**) Firstly, in response to EGF activation of HER2 signaling, cytoplasmic HNMT translocates to the cell membrane, and the HNMT/HER2 complex can be observed on the cell membrane within approximately 10–20 min (red arrows). Following this, (**b-c**) HNMT facilitates the recruitment of γ-secretase, which occurs around 30 min. Subsequently, the γ-secretase (PS1/PS2) complex forms with HER2, taking approximately 30–60 min. During this process, the HER2-ICD enters the cytoplasm (white arrowhead). (**d-e**) After 60 min of EGF treatment, it is observable that the HNMT/HER2-ICD complex enters the cell nucleus (yellow arrow). It binds to the HER2 promoter, inducing the upregulation of HER2 and HER2-associated oncogenic proteins. The newly synthesized HER2 protein subsequently translocates to the cell membrane, contributing to the manifestation of an H-cell phenotype and enhancing the binding of therapeutic agents such as trastuzumab or T-Dxd in HER2 + cells. **B** Based on the above results, the molecular mechanism of HNMT involvement in HER2 gene activation can be simplified, as shown in the schematic diagram

Based on the research findings above, it is evident that HNTM may interact with the HER2 protein, leading to the translocation of HER2-ICD into the nucleus and subsequent activation of the *HER2* gene. We have designed the experiment to treat SKBR3 cancer cells with EGF (100 ng/mL) and observe the kinetic time course of protein–protein complex formation. The results are shown in Fig. 5A. Upon EGF treatment, we observed that HNMT binds to HER2-ICD within a short period (~ 20 min), followed by the binding of γ-secretase to the HNMT/HER2 complex within 30–60 min, leading to the dissociation of HNMT/HER2-ICD into the cytoplasm. Subsequently (60–240 min), we observed the translocation of HNMT/HER2-ICD into the nucleus for the transcriptional regulation of the *HER2* gene. The schematic diagram in Fig. 5B simplifies and explains such a molecular mechanism. Our findings indicate that HNMT is selectively upregulated in HER2 + BCs and functions as an oncoprotein with two pivotal roles. First, our study revealed a novel mechanism through which HNMT overexpression, termed H-cells, fosters tumor proliferation by modulating the expression of the HER2 protein. It plays a role in the transcriptional activation of the HER2 protein. It augments the binding of trastuzumab and T-Dxd. Second, the H-cell phenotype can serve as a valuable diagnostic indicator for identifying individuals likely to respond effectively to anti-HER2 therapy.

## Discussion

Based on a previous report, the histamine level is higher in the plasma and cancer tissue of BC patients than in that of individuals without BC, mainly due to an imbalance in histamine synthesis and degradation [48–50]. HNMT is a crucial cytoplasmic enzyme that metabolizes histamine to N-methylhistamine in the airways and central nervous system [51, 52]. A previous study showed that HNMT can translocate from the cytoplasm to the plasma membrane upon receptor-mediated stimulation [30]. These results suggest that the rapid membrane translocation of HNMT is involved in biological functions related to histamine degradation rather than carcinogenesis. However, studies of single-membrane oncoproteins in cancer cells are insufficient to understand how tumor cell growth is altered or responds to therapeutic agents [53]. Previous studies by other groups [54] and our group [35] have demonstrated that PPIs between oncogenic proteins influence functional activity, structural stability, and intracellular localization. The importance of these PPI-induced accelerations of cancer cell growth and altered drug sensitivity is underestimated [25]. Therefore, monitoring the protein community associated with the overall drug treatment outcome is necessary.

Our previous paper presented a systems-integrated approach to generate the Cancer Membrane Protein Regulatory Network Resources ( http://campnets.life.nctu.edu.tw) containing 63,746 new PPIs of 1,962 membrane proteins using data from 5,922 expression profiles of individual tumors and overall survival outcomes for 15 human cancers [35]. For example, α9-nicotine acetylcholine receptor (α9-nAChR) [34], a membrane protein detected in smoking-related advanced BC patients, is associated with poorer survival outcomes. Using this platform, we discovered that the α9-nAChR forms complexes with 13 novel membrane proteins, including HER2. We demonstrated that a low dose of nicotine (200 nM) [34] binds to α9-nAChR and promotes its dissociation from HER2 [35] for clinical application. These results suggest that secondhand smoke and low-level nicotine exposure block the interaction between α9-nAChR and HER2 and activate HER2-mediated cancer formation [35]. Therefore, based on the characterization of protein-drug interaction profiles, we validated the potential of the FDA-approved drug bupropion as an antimetastatic agent for BC [35].

This study showed that in the presence of EGF in the tumor microenvironment, the HNMT and HER2 proteins can form a complex, leading to the shedding of the cytosolic HER2-ICD domain. A recent study showed that β2-adrenergic receptor activation promotes cleaved HER2-ICD formation and nuclear translocation [55] However, the proteolytic cleavage of HER2 by γ-secretase has not been well investigated [56]. Mechanistic studies suggest that shedding cytoplasmic RTK fragments generated by γ-secretase cleavage of full-length receptors is essential for initiating growth factor-mediated activation of downstream oncogenic signaling [57]. In addition, numerous studies have confirmed that nuclear HER2 can act as a coactivator or transcription factor to activate downstream genes, such as *cyclin D1* [58] and *COX-2* [45]. In addition, Yanli Bi et al. demonstrated that nuclear HER2 binds to the *DEPTOR* promoter to repress gene expression [59]. These results suggest that increasing HER2 protein levels in HER2 + BC cells via HER2-ICD-mediated transcription is critical for enhancing trastuzumab binding [55]. We found that the formation of HNMT/HER2-ICD complexes in tumor tissues (defined as H-cells) is sensitive to trastuzumab and T-Dxd treatment (Additional files 5 and 7: Fig. S5 and S7).

This issue holds significant importance due to the annual diagnosis of more than 287,000 new cases of BC in the United States. Among these patients, approximately 80% to 85% were HER2-negative (IHC score < 2 + and IHC score 2 + /ISH negative), with approximately 60% falling into the category of HER2-low BC patients. Consequently, there is a critical need to advance the development of specific diagnostic markers and targeted drugs tailored to this particular patient group. T-Dxd is an ADC that effectively targets tumor cells expressing low levels of HER2 [43, 60]. T-Dxd remains stable in the bloodstream and effectively targets tumor cells. Once inside the tumor, it is cleaved by cathepsins, releasing the payload to induce a cytotoxic effect on neighboring tumor cells. This targeted approach shows potential for treating cancer [61]. Based on a previous study, a phase 3 clinical trial involving patients with HER2-low metastatic BC was conducted [43]. In this study, the median progression-free survival (9.9 months, 95% CI, 9.0 to 11.3) in the T-Dxd arm was better than that in the physician's choice arm (5.1 months, 95% CI, 4.2 to 6.8) in the DESTINY-Breast04 trial (hazard ratio for disease progression or death, 0.50; 95% CI, 0.40 to 0.63; *P* < 0.001). T-Dxd significantly prolongs progression-free survival and overall survival in patients with HER2-low metastatic BC. This result suggested that an unknown factor is associated with a favorable prognosis with anti-HER2 therapy (trastuzumab and T-Dxd).

Our findings offer potential explanations for the outcomes observed in recent clinical trials [43] and provide molecular evidence supporting the association between HNMT expression and favorable prognosis in patients receiving anti-HER2 therapy for both HER2 + and HER2-low tumors. Specifically, our research demonstrated that the enforced overexpression of HNMT led to increased trastuzumab binding in various TNBC cell lines. Furthermore, we elucidated the role of the H-cell phenotype as a prognostic indicator for anti-HER2 therapy in HER2 + and HER2-low patients. For HER2 + BC patients exhibiting the H-cell phenotype, our use of FRET technology [35] to observe the formation of HNMT/HER2 complexes has shown promise in predicting a positive response to anti-HER2 treatment (Fig. 2I). Our findings indicate that patients with negative FRET results may experience relapse after multiple years of treatment. Consequently, it is imperative to proactively devise novel treatment approaches for these individuals to mitigate the risk of relapse. According to our research, H-cell phenotypes may help identify HER2-low (TNBC) BC patients who could benefit from anti-HER2 treatments such as T-Dxd [43]. This discovery offers the potential for developing more effective treatment protocols and improving patient survival rates for clinicians. This theory is supported by in vivo studies in mouse tumor models showing a significant response of H-cell-phenotype tumor cells to trastuzumab and T-Dxd treatments in HER2-low mouse tumor models (PDX and 231-HN, respectively) (Fig. 2F-K).

The ChIP-exo method was used in a recent study to uncover nuclear HER2 (CGT-TGC) binding sequences after EGF treatment in HER2 + BCLs (SKBR3 and BT474) [62]. The results revealed a common motif for the chromatin binding machinery of HER2, with de novo motif analysis indicating a motif shared across all binding peaks, resembling that in SKBR3 cells. Previous research pinpointed a HER2-associated binding sequence (TCAATTTC) in the *COX2* gene promoter [45]. This study (Table 3) also identified HNMT/HER2-ICD-induced HER2 amplicons [63–66], highlighting the critical role of HNMT-induced HER2-regulated downstream gene clusters. These findings emphasize the importance of HNMT/HER2-ICD-induced HER2 amplicons in regulating downstream gene clusters.

## Conclusions

Our research provides a comprehensive understanding of how HER2 amplicon expression is activated in HNMT-overexpressing cells, shedding light on the molecular basis for the distinctive H-cell phenotype observed in the tumor tissues of individuals responding to trastuzumab treatment. This highlights the potential of HNMT as a secondary marker for HER2 + BC, enhancing the accuracy of identifying patients who may benefit from trastuzumab therapy. Specific indicators are essential for successful therapies, particularly in patients with low HER2 expression. Our investigation revealed that HNMT is a potential prognostic marker for identifying individuals who may benefit from T-Dxd therapy.

## Supplementary Information

Additional file 1: Fig. S1 *HNMT* mRNA expression in the CCLE and TCGA databases and human breast tissue samples. (A) Correlation of *HNMT* with *HER2* expression in the CCLE database. (B-C) Quantitative *HNMT* mRNA expression in tumor tissues from different subtypes in the CCLE (B) and TCGA databases (C). (D) The *HNMT* mRNA expression profiles of paired human breast tumor (red lines) and normal (green lines) tissues were identified using real-time PCR. (E) Representative images from each step of LCM were illustrated. Green arrows indicate laser-imprinted normal cells; red arrows indicate tumor cells. Scale bars = 200 μm. Data are presented as the mean ± SE. Nonlinear regression, Pearson correlation analysis (A), and a two-tailed Mann‒Whitney U test (B-C) were performed for statistical analysis. *P < 0.05, **P < 0.01, and ***P < 0.001.

Additional file 2: Fig. S2 IHC histo-scoring of HNMT and HER2 protein expression in human BC TMAs. (A) Representative IHC images for HNMT and HER2 tissue scoring analysis. Scale bar = 100 μm. (B) Correlation of HNMT with the HER2 tissue score in human BC TMA samples (*n *= 211). Nonlinear regression and Pearson correlation analysis were performed to analyze tissue scores statistically. (C) Schematic representation of IHC tissue scores from tumor histotype cohorts (HER2+, TNBC, and luminal patients). The case number axis is shown on the x-axis to identify tissue groups. Each case included 1-4 tissue cohorts in the BC tumor TMAs. Different colors are indicated in blocks based on differences in HNMT and HER2 histo-scores. (D-E) The overall survival of BC patients was analyzed using the Kaplan‒Meier method, with the patients grouped based on their *HNMT *mRNA expression levels (D) and the combination of *HNMT* and *HER2* mRNA expression levels (E). (F) The inhibitory effect of the HNMT inhibitor SKF-91488 on cancer cell growth was tested in different cell lines in a dose-dependent manner. Nonlinear regression, Pearson correlation analysis (B), a log-rank test (D, E), and a two-tailed unpaired Student's t-test (F) were performed for statistical analysis.

Additional file 3: Fig. S3 In vitro and in vivo carcinogenic potential of HNMT in BC cells. (A-B) MDA-MB-231 cells harboring HNMT siRNA (A) or overexpressing HNMT (B) were subjected to cell viability, migratory wound healing, and anchorage-independent growth soft agar assays. Invasion assay in MDA-MB-231 cells overexpressing HNMT (B). The irregular red lines in the migration assay images indicate wound healing areas. Western blot analysis confirmed the expression of the indicated proteins in MDA-MB 231 cells (231-Wt) and stable cells (231-Vc, 231-HN) (B). (C) On the left are bioluminescence images of 231-Wt-Luc, 231-Vc-Luc, and 231-HN-Luc tumor-bearing mice. Lung and tumor tissues from 231-Vc and 231-HN mice were collected after sacrifice for H&E staining to observe and quantify metastatic nodules. The representative H&E staining image on the right includes high-magnification images of 231-Vc and 231-HN tumor tissues highlighted in blue and red boxes, respectively. (D-E) Western blot analysis confirmed the expression of the indicated proteins in MDA-MB 231 cells (231-Wt), stable cells (231-Vc, 231-HN), primary cells derived from xenografted tumors (231-Vc-XE, 231-HN-XE), metastatic lung cancer cells (231-HN-LM) (D), and 231-HN cells harboring HNMT-scramble or HNMT-siRNA (E). (F) 231-HN and 231-Vc cells underwent RNA sequencing analysis (lower panel). We treated these cells with an E2F1-specific inhibitor (HLM006474, 40 μM for 24 h) and performed Western blot analysis to analyze the protein expression of T-AKT, using actin as a loading control (picture below). (G) We treated cells overexpressing HNMT (231-HN) and control cells (231-Vc) with EGF (100 ng/mL) for 24 h, and then performed RNA-Seq analysis. This data was consistent with previously published Genomic comparisons of PEDERSEN gene sets activated by PI3K/Akt or MAPK pathways in HER2+ tumors (NES = 1.24, *P* value = 0.04). The data are the means ± SEs. Statistical analysis was performed using a two-tailed unpaired Student's t-test. **P < 0.01 and ***P < 0.001. Scale bars = 250 µm (A, B) and 2 mm (C).

Additional file 4: Fig. S4 HNMT expression affected the binding affinity of TNBC tumor cells for trastuzumab. (A) Western blot analysis of HNMT and HER2 protein expression in SKBR3 cells transfected with the vector or with the HNMT siRNA alone, the HER2 siRNA alone, or the combined HNMT/HER2 siRNA. (B) Schematic diagram of the timeline for DOX (5 μg/mL)-induced HNMT expression via the T-REx^TM^ system (pcDNA5/TO-HNMT and pcDNA6/TR vectors) at time-dependent manner (0, 24, and 48 h). (C-D) Western blotting (C) and flow cytometric analysis (D) are shown. (E) Western blotting confirmed the protein expression of HNMT and HER2 in TNBC cells harboring the vector or overexpressing HNMT. (F) In the above cells (E), flow cytometry was used to detect the binding affinity of trastuzumab (0.1, 0.5, 1 μg/mL) for the HER2 protein. *n *= 3 biologically independent experiments. The data are presented as the means ± SEs. Statistical analysis was performed using a two-tailed unpaired Student's t-test. *P < 0.05, **P < 0.01, and ***P < 0.001.

Additional file 5: Fig. S5 H- and L-cell phenotypes visualized by IHC staining in BC tumor tissue. (A-C) H&E , HER2 IHC, and HNMT/HER2 dual IHC staining of HER2+ BC tumor tissues (A), PDX tumors (B), and HNMT-overexpressing TNBC cell xenograft tumors (MDA-MB-231 (top) and HCC38 (bottom)) (C). Scale bar = 20 µm.

Additional file 6: Fig. S6 FRET spectrum and trastuzumab responders versus non-responders in HER+ BC patients. (A) In the GSE50948 microarray dataset, HNMT protein expression was compared between the pathological complete response (pCR) and residual disease (RD) groups. The results revealed distinct expression levels, with the pCR group showing a notable increase in HNMT protein expression (depicted in red) compared to the RD group (depicted in black). The data are presented as the means ± SE. Statistical analysis was performed using the two-tailed Mann‒Whitney U test. (B-C) FRET images of HNMT and HER2-ICD protein interactions in the tumor tissues of BC patients who were trastuzumab therapy responders (*n* = 41) (B) and non-responders (*n* = 7) (C). Scale bar = 10 µm.

Additional file 7: Fig. S7 The upregulation of HNMT expression, also referred to as the H-cell phenotype, enhances the in vivo binding affinity of T-Dxd. (A) The IC_50_ values of T-Dxd treatment (0, 0.01, 0.1, 0.5, 1, 5, 10, 25, and 50 μg/mL) were detected in HER2+ (HCC1954) and TNBC (231-wt, 231-Vc, and 231-HN) cell lines. (B) The time-dependent (0, 0.5, 1, 4, 8, 16 h) binding affinity of T-Dxd (10 μg/mL) was observed by flow cytometry (top) and IF staining (bottom) in HCC1954 cells. The yellow box is an enlarged image. The white arrows indicate T-Dxd located in the lysosome. The data are presented as the means ± SE. Statistical analysis was performed using a two-tailed unpaired Student's t-test. ***P < 0.001. (C) H&E and IHC were performed on TNBC (231-Vc and 231-HN) xenograft tumor tissues. The tumors were treated with/without T-Dxd (4 mg/kg). *n* =4 biologically independent experiments. A schematic diagram showing that T-Dxd-induced HNMT expression (H-cell phenotype) in vivo enhances T-Dxd binding affinity, resulting in tumor regression. (D) Western blotting confirmed the T-Dxd-induced expression of HNMT (H-cell phenotype) in MDA-MB-231 cells. Actin protein was used as a loading control. (E) Tumor tissue obtained from Fig. 2K was used for the ex vivo T-Dxd binding affinity assay. Representative fluorescence images of T-Dxd-treated *ex vivo* tumors. Yellow arrows indicate T-Dxd binding to 231-Vc- and 231-HN-derived xenograft tumors. This schematic shows that T-Dxd-induced HNMT (the L-cell phenotype transforms to the H-cell phenotype) in 231-Vc-derived xenograft tumors (in vivo) enhances the binding affinity of T-Dxd. Scale bars = 18.4 μm (B, C) and 100 μm (E).

Additional file 8: Fig. S8 Receptor-mediated interactions between HER2 and HNMT in BC cells. (A) BC cells were treated with EGF (100 ng/mL) in a time-dependent manner (0, 10, 20,30, and 60 min), and immunoblot analysis revealed HNMT protein expression in subcellular fractions. (B) Proteins isolated from EGF-treated SKBR3 cells in a time-dependent manner (0, 20, and 50 min), and the cells were immunoprecipitated to observe the interaction between the HNMT, HER1 and HER2 proteins. (C) Schematic representation of the ligand-mediated interaction between the HNMT and HER2 proteins at the cell membrane (left) at 20 min. Representative images and quantitative results of FRET efficiency in SKBR3 cells treated with and without EGF or NRG (100 ng/mL and 100 μM, respectively). Yellow arrows indicate a positive FRET signal. The data are presented as the means ± SE. Statistical analysis was performed using a two-tailed unpaired Student's t-test. ***P < 0.001. (D) The Supplementary Data of Fig. 3A shows schematic representations of molecular interactions and IF images with positive (right) or negative (left) FRET signals. (E) After treating SKBR3 cells with EGF (100 ng/mL) for 20 min, the FRET technique was employed to analyze the potential interaction between HER1 and HNMT. (F) SKBR3 cells were transiently transfected with CRISPR control and HER1 KO plasmids. Western blot analysis confirmed the expression of the indicated proteins in SKBR3 stable cells (SK-Vc and SK-HN). After the cells were treated with and without EGF (100 ng/mL) for 20 min, the interaction between HNMT and HER2 protein on the cell membrane was observed. It shows that the interaction between HNMT/HER2 is obvious in the presence of EGF (red arrow). Without HER1 protein, the interaction between HNMT and HER2 protein is blocked. Scale bar=10 µm (C, D, E, F).

Additional file 9: Fig. S9 HNMT is involved in γ-secretase-induced fragmentation of the HER2 protein, leading to cytosolic HER2-ICD shedding. (A) Schematic diagram of the γ-secretase-mediated enzymatic cleavage site in the HER2 protein sequence [41]. (B) Schematic illustration of HER2-ICD cleavage by HNMT-activated γ-secretase (left). Western blotting confirmed the expression of the indicated proteins in CHX-treated (20 μg/mL) SK-Vc, SK-HN, SK-Sc, and SK-Si cells (right) in a time-dependent manner (0, 24, and 48 h). (C) SK-Vc, SK-HN, SK-Sc, and SK-Si cell lysates were immunoprecipitated for HNMT and immunoblotted for relevant proteins as indicated. (D)Western blotting confirmed protein expression in SKBR3 cells containing scrambled PS1/2 and PS1/2 siRNA treated with or without EGF (100 ng/mL) for 24 h. (E) The expression of the indicated proteins in SKBR3 cells treated with or without CHX (20 μg/mL) at 24 h and containing scrambled PS1 and PS2 sequences, PS1 siRNA, or PS2 siRNA was confirmed by Western blotting.

Additional file 10: Fig. S10 HNMT expression affects the binding of HER2+ tumor cells to trastuzumab. (A) The cellular localization of different parts of the HER2 protein was confirmed by IF staining. Schematic representation of regions recognized by trastuzumab and anti-HER2-ICD antibodies (left). Representative images and qanitification data of IF double-stained cells showing the cellular distribution of HNMT and HER2 isoforms by 100 ng/mL EGF treating 20 min. The right panel shows HER2-ICD detection using the HER2-ICD antibody (green) and p185HER2 detection using trastuzumab (green). The white arrows indicate the nuclear localization of HER2-ICD, while the yellow arrows indicate negative results. *n*=5 biologically independent experiments. Scale bar = 10 µm. (B) The frequencies of the top 50 ranked target genes were determined by ChIP-sequencing analysis. (C) pGL3-4X-HBS-1 and pGL3-4X-HBS-2 were transfected into SKBR3 cells expressing the HER2 scramble or HER2 siRNA, respectively. Quantitative luc reporter assays were performed following the treatment of transfected cells with/without EGF or NRG1. The pGL3 vector was transfected into SKBR3 cells as a control. The pRL-TK plasmid was used as an internal control. (D) Western blotting confirmed HNMT and HER2 protein expression in the HER2+ BC cell lines harboring vectors overexpressing HNMT, HNMT scrambled RNA or HNMT siRNA. (E) The ability of trastuzumab to bind to the HER2 protein in the above four cell lines (D) was detected by flow cytometry. *n *= 3 biologically independent experiments. The data are presented as the mean ± SE. Statistical analysis was performed using a two-tailed unpaired Student's t-test. *P < 0.05, **P < 0.01, and ***P < 0.001.

Additional file 11: Fig. S11 Genetic background verification of PDX tumor tissue based on RNA sequencing data. Reference heatmaps of RNA sequencing data from both JAX and our laboratory are available for comparison. The colored lines represent individual genes. Consistent gene expression is depicted in the heatmap.

Additional file 12.

## Data Availability

The data supporting this study's findings are available within the article and its supplementary materials. Researchers and interested parties can access the relevant information and supporting evidence from the corresponding authors upon reasonable request. The data of HNMT mRNA expression (Public 23Q2) that support the findings of this study are openly available in CCLE at https://sites.broadinstitute.org/ccle/. The HNMT mRNA expression and clinical information data are available under the TCGA database (https://portal.gdc.cancer.gov/). Detailed clinicopathological information and RNA sequencing data of PDX tumors are available from JAX: https://www.cancermodels.org/data/models/JAX/J000100674 and J000110056: https://www.cancermodels.org/data/models/JAX/J000111056. The RNA-seq data generated in this study can be obtained in GEO, the accession number is (GSE262820, review password: ifwhegskllipfaj). Additional data supporting the findings of this study are available from the corresponding author upon request.

## Abbreviations

ADC: Antibody‒drug conjugate
ATCC: American Type Culture Collection
BC: Breast cancer
CCLE: Cancer cell line encyclopedia
CHX: Cycloheximide
ChIP: Chromatin immunoprecipitation
DOX: Doxycycline
ECD: Extracellular domain
EGF: Epidermal growth factor
FLIM: Fluorescence lifetime imaging microscopy
FRET: Förster resonance energy transfer
HAPs: HNMT-associated proteins
HBS: HNMT binding site
HER2 +: Human epidermal growth factor receptor 2-positive
HNMT: Histamine N-methyltransferase
KO: Knocked-out
LCM: Laser capture microdissection
LM: Lung metastasis
Luc: Luciferase
ICD: Intracellular domain
IF: Immunofluorescence
IHC: Immunohistochemistry
IP: Immunoprecipitation
NLS: Nuclear localization signal
NRG1: Neuregulin-1
pCR: Pathological complete response
PDX: Patient-derived xenograft
PPI: Protein–protein interaction
PS1: Presenilin 1
PS2: Presenilin 2
RD: Residual disease
RTKI: Receptor tyrosine kinase inhibitor
TCGA: The Cancer Genome Atlas
T-Dxd: Trastuzumab deruxtecan
T-DM1: Trastuzumab emtansine
TMA: Tissue microarray
TNBC: Triple negative breast cancer
