# Correction: Histamine N-methyltransferase (HNMT) as a potential auxiliary biomarker for predicting adaptability to anti-HER2 drug treatment in breast cancer patients

**DOI:** 10.1186/s40364-025-00819-6

**Published:** 2025-08-07

**Authors:** Tzu-Chun Cheng, Mien-Chie Hung, Lu-Hai Wang, Shih-Hsin Tu, Chih-Hsiung Wu, Yun Yen, Chi-Long Chen, Jacqueline Whang-Peng, Wen-Jui Lee, You-Cheng Liao, Yu-Ching Lee, Min-Hsiung Pan, Hui-Kuan Lin, Huey-En Tzeng, Peixuan Guo, Cheng-Ying Chu, Li-Ching Chen, Yuan-Soon Ho

**Affiliations:** 1 Institute of Biochemistry and Molecular Biology, College of Life Sciences, China Medical University, Taichung, Taiwan; 2 Graduate Institute of Biomedical Sciences, Institute of Biochemistry and Molecular Biology, Research Center for Cancer Biology, Cancer Biology and Precision Therapeutics Center, and Center for Molecular Medicine, China Medical University, Taichung, Taiwan; 3 Department of Biotechnology, Asia University, Taichung, Taiwan; 4 Institute of Integrated Medicine and Chinese Medicine Research Center, China Medical University, Taichung, Taiwan; 5 Department of Surgery, School of Medicine, College of Medicine, Taipei Medical University, Taipei, Taiwan; 6 Department of Surgery, Taipei Medical University Hospital, Taipei, Taiwan; 7 TMU Research Center of Cancer Translational Medicine, Taipei Medical University, Taipei, Taiwan; 8 Department of Pathology, School of Medicine, College of Medicine, Taipei Medical University, Taipei, Taiwan; 9 Department of Pathology, Taipei Medical University Hospital, Taipei Medical University, Taipei, Taiwan; 10 Ph.D. Program in Medical Neuroscience, College of Medical Science and Technology, Taipei Medical University, Taipei, Taiwan; 11 Institute of Food Sciences and Technology, National Taiwan University, Taipei, Taiwan; 12 Department of Pathology, Duke University Medical Center, Duke University School of Medicine, Durham, NC 27710 USA; 13 Division of Hematology/Medical Oncology, Department of Medicine, Taichung Veterans General Hospital, Taichung City, Taiwan; 14 Department of Post-Baccalaureate Medicine, College of Medicine, National Chung-Hsing University, Taichung, Taiwan; 15 Center for RNA Nanobiotechnology and Nanomedicine, College of Pharmacy, College of Medicine, Dorothy M. Davis Heart and Lung Research Institute, and James Comprehensive Cancer Center, The Ohio State University, Columbus, OH 43210 USA; 16 CRISPR Gene Targeting Core, Taipei Medical University, Taipei, 110 Taiwan; 17 Department of Biological Science & Technology, College of Life Sciences, China Medical University, Taichung, Taiwan

**Correction: Biomarker Research 13, 7 (2025)**

**https://doi.org/10.1186/s40364-024–00715-5**

The authors discovered an issue in Fig. 5A in the original article whereby an editing error duplicated an image.

The authors have identified the original data files and confirmed the correct version of the figure which can be viewed ahead in this Correction article.

**Fig. 5:**
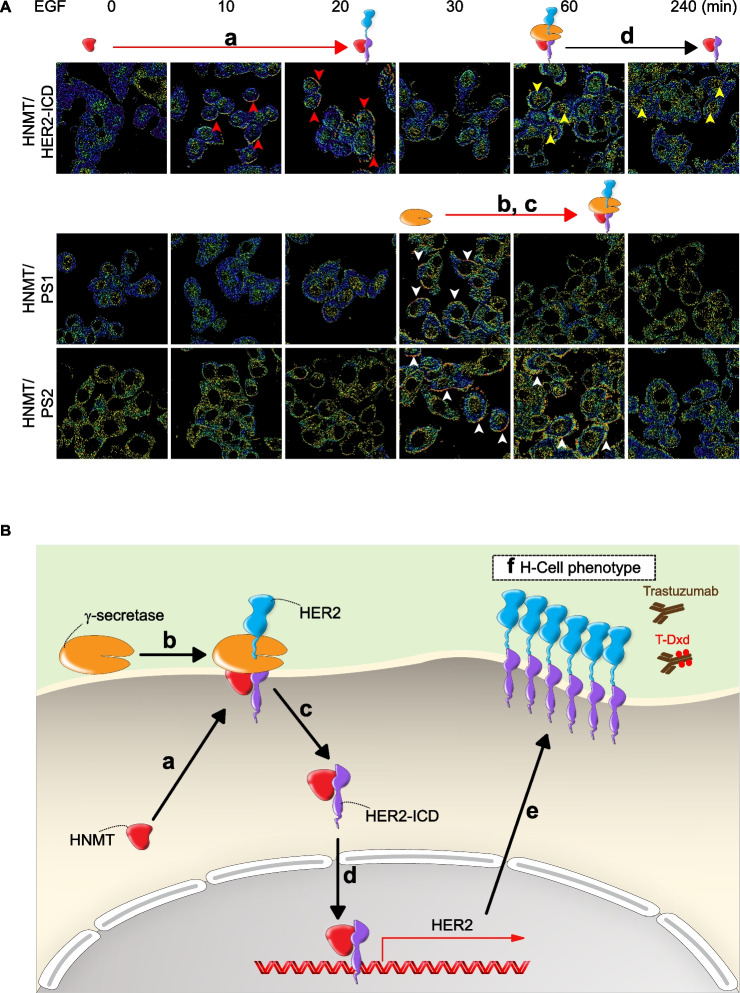
The molecular mechanism by which HNMT enhances BC cell sensitivity to anti-HER2 targeted drugs. The molecular mechanism underlying HER2-induced oncogenesis involves a series of intricate steps. **A** In this study, EGF (100 ng/mL) was added to SKBR3 cancer cells, and the kinetic process of complex formation was observed. (**a**) Firstly, in response to EGF activation of HER2 signaling, cytoplasmic HNMT translocates to the cell membrane, and the HNMT/HER2 complex can be observed on the cell membrane within approximately 10–20 min (red arrows). Following this, (**b**-**c**) HNMT facilitates the recruitment of γ-secretase, which occurs around 30 min. Subsequently, the γ-secretase (PS1/PS2) complex forms with HER2, taking approximately 30–60 min. During this process, the HER2-ICD enters the cytoplasm (white arrowhead). (**d**-**e**) After 60 min of EGF treatment, it is observable that the HNMT/HER2-ICD complex enters the cell nucleus (yellow arrow). It binds to the HER2 promoter, inducing the upregulation of HER2 and HER2-associated oncogenic proteins. The newly synthesized HER2 protein subsequently translocates to the cell membrane, contributing to the manifestation of an H-cell phenotype and enhancing the binding of therapeutic agents such as trastuzumab or T-Dxd in HER2+cells. **B** Based on the above results, the molecular mechanism of HNMT involvement in HER2 gene activation can be simplifed, as shown in the schematic diagram

