# Supplementary material for: Histamine N-methyltransferase (HNMT) as a potential auxiliary biomarker for predicting adaptability to anti-HER2 drug treatment in breast cancer patients
Source: Biomark Res. 2025 Jan 9;13:7. doi: 10.1186/s40364-024-00715-5 (PMC11720525; doi:10.1186/s40364-024-00715-5)
Supplement: Supplementary file 1 — Additional file 1: Fig. S1 HNMT mRNA expression in the CCLE and TCGA databases and human breast tissue samples. (A) Correlation of HNMT with HER2 expression in the CCLE database. (B-C) Quantitative HNMT mRNA expression in tumor tissues from different subtypes in the CCLE (B) and TCGA databases (C). (D) The HNMT mRNA expression profiles of paired human breast tumor (red lines) and normal (green lines) tissues were identified using real-time PCR. (E) Representative images from each step of LCM were illustrated. Green arrows indicate laser-imprinted normal cells; red arrows indicate tumor cells. Scale bars = 200 μm. Data are presented as the mean ± SE. Nonlinear regression, Pearson correlation analysis (A), and a two-tailed Mann‒Whitney U test (B-C) were performed for statistical analysis. *P < 0.05, **P < 0.01, and ***P < 0.001. [file 40364_2024_715_MOESM1_ESM.zip › Additional file 1.docx]

**Table S1:** Primers used in this manuscript.

| Oligonucleotides |
| --- |
| HNMT-specific primers for real-time PCR and RT‒PCR  F: 5’-CTACCCTGAAATTCTTCCATAGTCT  R: 5’-TGAGTGAGGTCATCTGATGTGATA |
| GUS-specific primers for real-time PCR and RT‒PCR  F: 5’-AAACAGCCCGTTTACTTGAG  R: 5’-AGTGTTCCCTGCTAGAATAGATG |
| HNMT ScRNA primer inserted into the pSUPER plasmid  F: 5’-GATCCCCCGAGCACATACACGTCTATTTCAAGAGAATAGACGTGTATGTGCTCGTTTTTA  R: 5’-AGCTTAAAAACGAGCACATACACGTCTATTCTCTTGAAATAGACGTGTATGTGCTCGGGG |
| HNMT SiRNA primer inserted into the pSUPER vector  F: 5’-GATCCCCAAGTTCAGGCTCAATACCCTTCAAGAGAGGGTATTGAGCCTGAACTTTTTTTA  R: 5’-AGCTTAAAAAAAGTTCAGGCTCAATACCCTCTCTTGAAGGGTATTGAGCCTGAACTTGGG |
| HER2 ScRNA primer inserted into the pSUPER vector  F: 5’-GATCCCCCGAGCACATACACGTCTATTTCAAGAGAATAGACGTGTATGTGCTCGTTTTTA  R: 5’-AGCTTAAAAACGAGCACATACACGTCTATTCTCTTGAAATAGACGTGTATGTGCTCGGGG |
| HER2 SiRNA primer inserted into the pSUPER vector  F: 5’-GATCCCCAAGTTCAGGCTCAATACCCTTCAAGAGAGGGTATTGAGCCTGAACTTTTTTTA  R: 5’-AGCTTAAAAAAAGTTCAGGCTCAATACCCTCTCTTGAAGGGTATTGAGCCTGAACTTGGG |
| PS1 ScRNA primer inserted into the pSUPER vector  F: 5’-GATCCCCGCTTCATAAGGCGCATAGCTTCAAGAGAGCTATGCGCCTTATGAAGCTTTTTA  R: 5’-AGCTTAAAAAGCTTCATAAGGCGCATAGCTCTCTTGAAGCTATGCGCCTTATGAAGCGGG |
| PS1 SiRNA primer inserted into the pSUPER vector  F: 5’-GATCCCCGATGAGGAGCTGACATTGATTCAAGAGATCAATGTCAGCTCCTCATCTTTTTA  R: 5’-AGCTTAAAAAGATGAGGAGCTGACATTGATCTCTTGAATCAATGTCAGCTCCTCATCGGG |
| HNMT overexpression primer inserted into the pcDNA5/TO vector  F: 5’-TTTAAGCTTATGGCATCTTCCATGAGGAG  R: 5’-TTTGGATCCTGCCTCAATCACTATGAAAC |
| HNMT primer inserted into the pcDNA5/TO-ZsYellow vector  F: 5’-TTTGTCGACATGGCATCTTCCATGAGGAG  R: 5’-TTTGGATCCTTATGCCTCAATCACTATGA |
| HER2 primer inserted into the pcDNA5/TO-AmCyan vector  F: 5’-AAAGCTAGCATGGAGCTGGCGGCCTTGTG  R: 5’-AAAAAGCTTCACTGGCACGTCCAGACCCA |
| 4X HBS-1 reporter primer inserted into the pGL3 vector  F: 5’-AAAACGCGTGATGGGGGCAAGGTTAGGTGATGGGGGCAAGGTTAGGTGATGGGGGCAAGGTTAGGTGATGGGGGCAAGGTTAGGTAGATCTAAA  R: TTTAGATCTACCTAACCTTGCCCCCATCACCTAACCTTGCCCCCATCACCTAACCTTGCCCCCATCACCTAACCTTGCCCCCATCACGCGTTTT |
| 4X HBS-2 reporter primer inserted into the pGL3 vector  F: 5’-AAAACGCGTGCCGGGGGAGGAGCTTAGGGCCGGGGGAGGAGCTTAGGGCCGGGGGAGGAGCTTAGGGCCGGGGGAGGAGCTTAGGAGATCTAAA  R: 5’-TTTAGATCTCCTAAGCTCCTCCCCCGGCCCTAAGCTCCTCCCCCGGCCCTAAGCTCCTCCCCCGGCCCTAAGCTCCTCCCCCGGCACGCGTTTT |
| HNMT primer inserted into the pcDNA3.1 N-Luc vector  F: 5’-AAAGCTAGCATGGCATCTTCCATGAGGAG  R: 5’-AAAAAGCTTTGCCTCAATCACTATGAAAC |
| HER2 primer inserted into the pcDNA3.1 C-Luc vector  F: 5’-AAAGCTAGCATGGAGCTGGCGGCCTTGTG  R: 5’-AAAAAGCTTCACTGGCACGTCCAGACCCA.  HNMT KO sgRNA primer  F: 5’- TGACTCAGTAATGAGCTCTACAAGCCAGCCT  R: 5’- GCTATCAGTAATTCGCCCTTATCCCTGTGC  HER1 KO sgRNA primer  F：5’-CACCGTGAGCTTGTTACTCGTGCCT  R：5/-AAACAGGCACGAGTAACAAGCTCAC |
