# Supplementary figures and images for: Histamine N-methyltransferase (HNMT) as a potential auxiliary biomarker for predicting adaptability to anti-HER2 drug treatment in breast cancer patients
Source: Biomark Res. 2025 Jan 9;13:7. doi: 10.1186/s40364-024-00715-5 (PMC11720525; doi:10.1186/s40364-024-00715-5)

**Fig. S1**

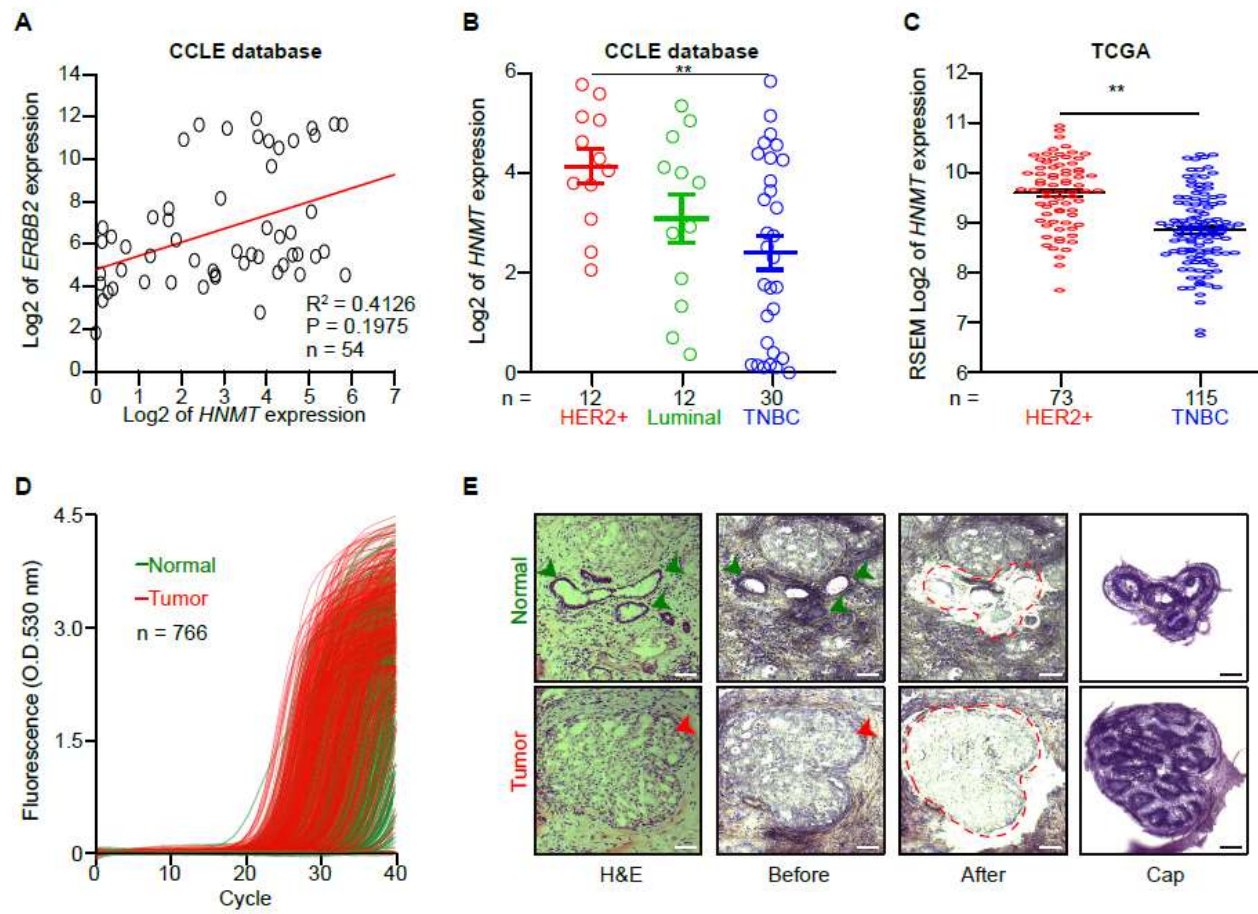

Supplement: Supplementary file 1 — Additional file 1: Fig. S1 HNMT mRNA expression in the CCLE and TCGA databases and human breast tissue samples. (A) Correlation of HNMT with HER2 expression in the CCLE database. (B-C) Quantitative HNMT mRNA expression in tumor tissues from different subtypes in the CCLE (B) and TCGA databases (C). (D) The HNMT mRNA expression profiles of paired human breast tumor (red lines) and normal (green lines) tissues were identified using real-time PCR. (E) Representative images from each step of LCM were illustrated. Green arrows indicate laser-imprinted normal cells; red arrows indicate tumor cells. Scale bars = 200 μm. Data are presented as the mean ± SE. Nonlinear regression, Pearson correlation analysis (A), and a two-tailed Mann‒Whitney U test (B-C) were performed for statistical analysis. *P < 0.05, **P < 0.01, and ***P < 0.001. [file 40364_2024_715_MOESM1_ESM.zip › Additional file 1.pdf]

Fig. S2

A

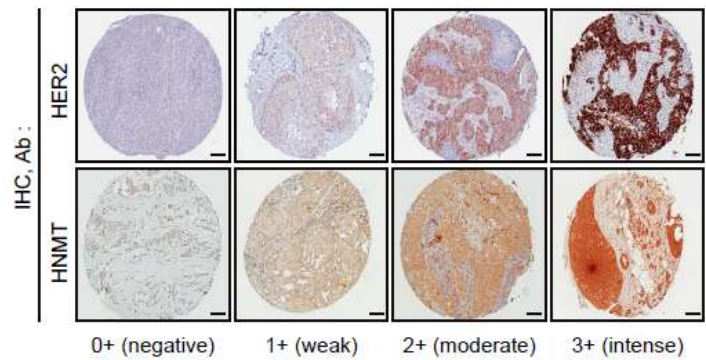

B

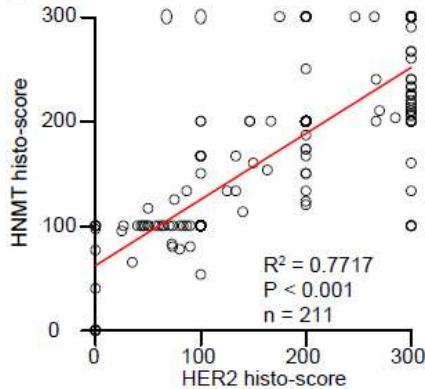

C

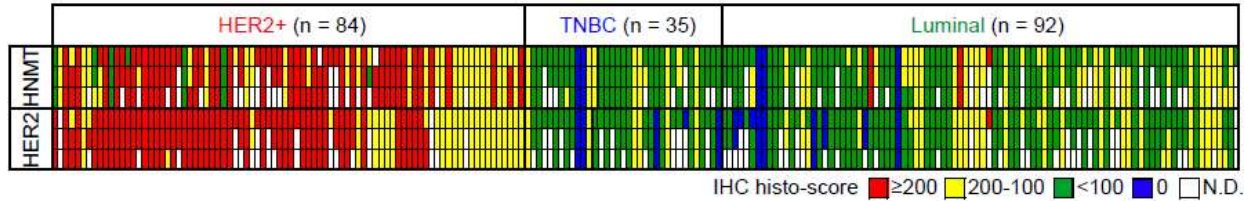

D

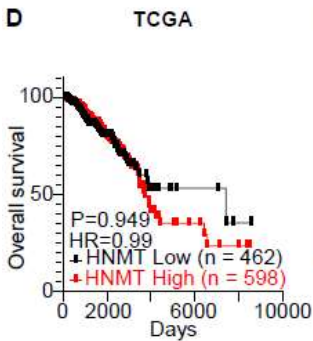

E

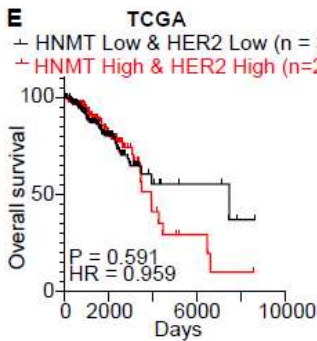

F

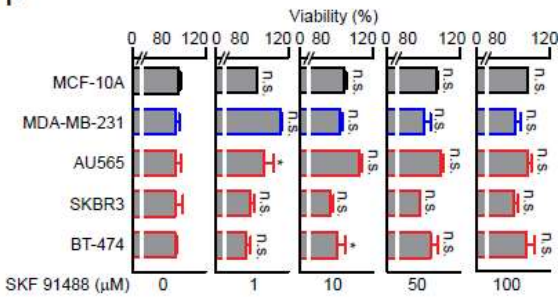

Supplement: Supplementary file 2 — Additional file 2: Fig. S2 IHC histo-scoring of HNMT and HER2 protein expression in human BC TMAs. (A) Representative IHC images for HNMT and HER2 tissue scoring analysis. Scale bar = 100 μm. (B) Correlation of HNMT with the HER2 tissue score in human BC TMA samples (n = 211). Nonlinear regression and Pearson correlation analysis were performed to analyze tissue scores statistically. (C) Schematic representation of IHC tissue scores from tumor histotype cohorts (HER2+, TNBC, and luminal patients). The case number axis is shown on the x-axis to identify tissue groups. Each case included 1-4 tissue cohorts in the BC tumor TMAs. Different colors are indicated in blocks based on differences in HNMT and HER2 histo-scores. (D-E) The overall survival of BC patients was analyzed using the Kaplan‒Meier method, with the patients grouped based on their HNMT mRNA expression levels (D) and the combination of HNMT and HER2 mRNA expression levels (E). (F) The inhibitory effect of the HNMT inhibitor SKF-91488 on cancer cell growth was tested in different cell lines in a dose-dependent manner. Nonlinear regression, Pearson correlation analysis (B), a log-rank test (D, E), and a two-tailed unpaired Student's t-test (F) were performed for statistical analysis. [file 40364_2024_715_MOESM2_ESM.pdf]

Fig. S3

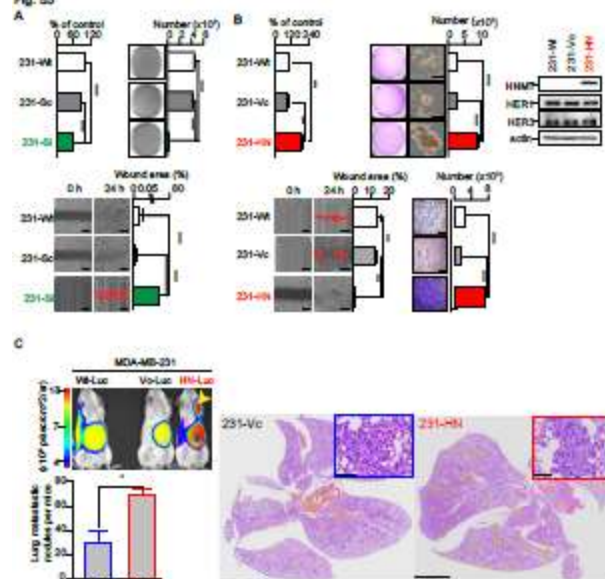

Fig. S3

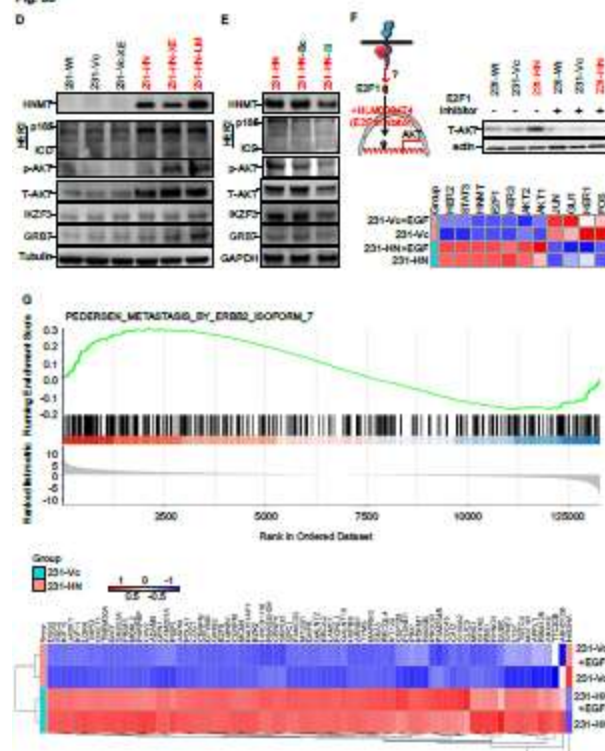

Supplement: Supplementary file 3 — Additional file 3: Fig. S3 In vitro and in vivo carcinogenic potential of HNMT in BC cells. (A-B) MDA-MB-231 cells harboring HNMT siRNA (A) or overexpressing HNMT (B) were subjected to cell viability, migratory wound healing, and anchorage-independent growth soft agar assays. Invasion assay in MDA-MB-231 cells overexpressing HNMT (B). The irregular red lines in the migration assay images indicate wound healing areas. Western blot analysis confirmed the expression of the indicated proteins in MDA-MB 231 cells (231-Wt) and stable cells (231-Vc, 231-HN) (B). (C) On the left are bioluminescence images of 231-Wt-Luc, 231-Vc-Luc, and 231-HN-Luc tumor-bearing mice. Lung and tumor tissues from 231-Vc and 231-HN mice were collected after sacrifice for H&E staining to observe and quantify metastatic nodules. The representative H&E staining image on the right includes high-magnification images of 231-Vc and 231-HN tumor tissues highlighted in blue and red boxes, respectively. (D-E) Western blot analysis confirmed the expression of the indicated proteins in MDA-MB 231 cells (231-Wt), stable cells (231-Vc, 231-HN), primary cells derived from xenografted tumors (231-Vc-XE, 231-HN-XE), metastatic lung cancer cells (231-HN-LM) (D), and 231-HN cells harboring HNMT-scramble or HNMT-siRNA (E). (F) 231-HN and 231-Vc cells underwent RNA sequencing analysis (lower panel). We treated these cells with an E2F1-specific inhibitor (HLM006474, 40 μM for 24 h) and performed Western blot analysis to analyze the protein expression of T-AKT, using actin as a loading control (picture below). (G) We treated cells overexpressing HNMT (231-HN) and control cells (231-Vc) with EGF (100 ng/mL) for 24 h, and then performed RNA-Seq analysis. This data was consistent with previously published Genomic comparisons of PEDERSEN gene sets activated by PI3K/Akt or MAPK pathways in HER2+ tumors (NES = 1.24, P value = 0.04). The data are the means ± SEs. Statistical analysis was performed using a two-tailed unpaire [file 40364_2024_715_MOESM3_ESM.pdf]

Fig. S4

A

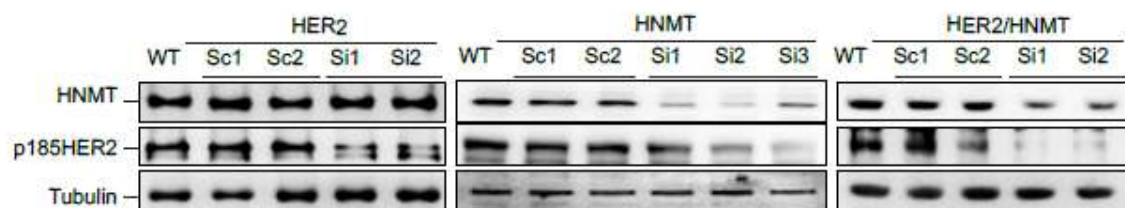

B

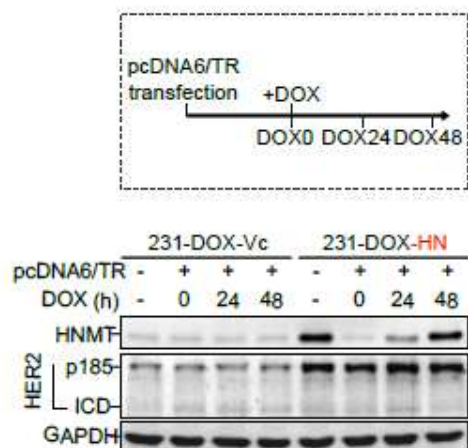

D

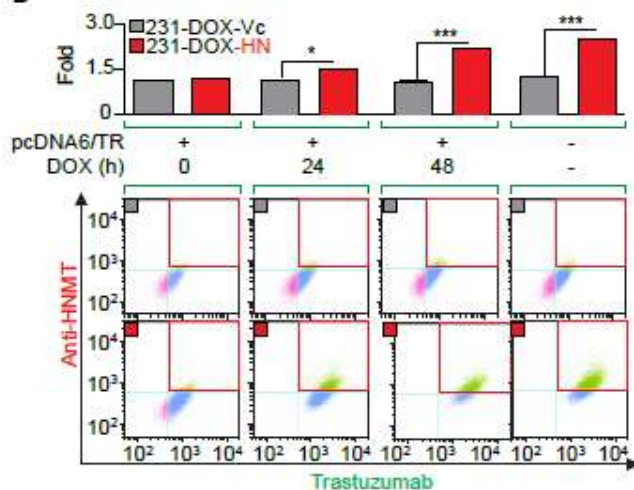

E

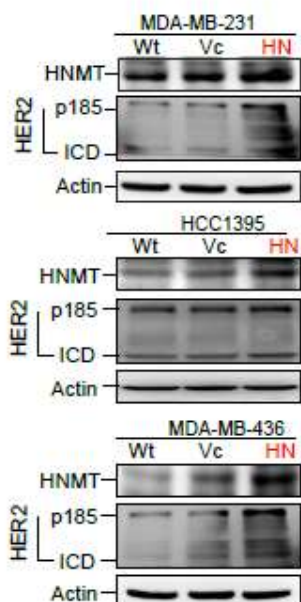

F

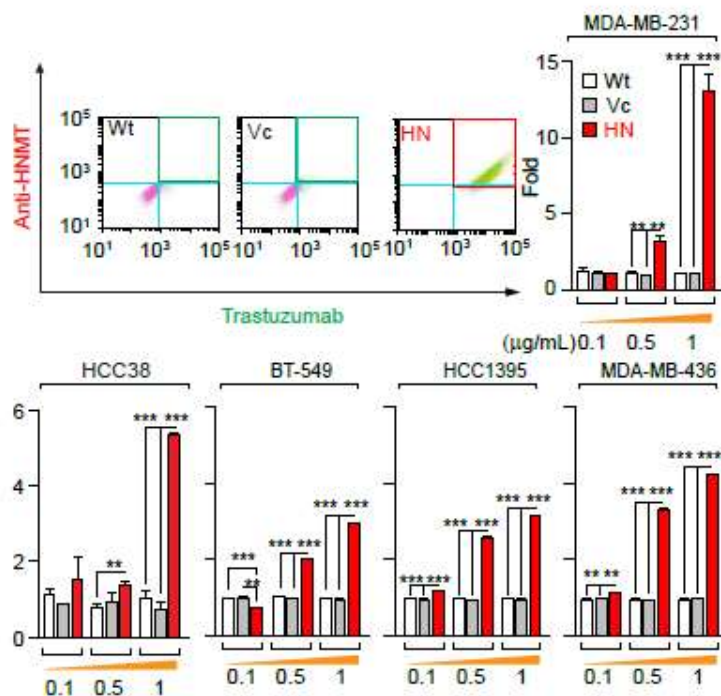

Supplement: Supplementary file 4 — Additional file 4: Fig. S4 HNMT expression affected the binding affinity of TNBC tumor cells for trastuzumab. (A) Western blot analysis of HNMT and HER2 protein expression in SKBR3 cells transfected with the vector or with the HNMT siRNA alone, the HER2 siRNA alone, or the combined HNMT/HER2 siRNA. (B) Schematic diagram of the timeline for DOX (5 μg/mL)-induced HNMT expression via the T-RExTM system (pcDNA5/TO-HNMT and pcDNA6/TR vectors) at time-dependent manner (0, 24, and 48 h). (C-D) Western blotting (C) and flow cytometric analysis (D) are shown. (E) Western blotting confirmed the protein expression of HNMT and HER2 in TNBC cells harboring the vector or overexpressing HNMT. (F) In the above cells (E), flow cytometry was used to detect the binding affinity of trastuzumab (0.1, 0.5, 1 μg/mL) for the HER2 protein. n = 3 biologically independent experiments. The data are presented as the means ± SEs. Statistical analysis was performed using a two-tailed unpaired Student's t-test. *P < 0.05, **P < 0.01, and ***P < 0.001. [file 40364_2024_715_MOESM4_ESM.pdf]

**Fig. S6**

**A**

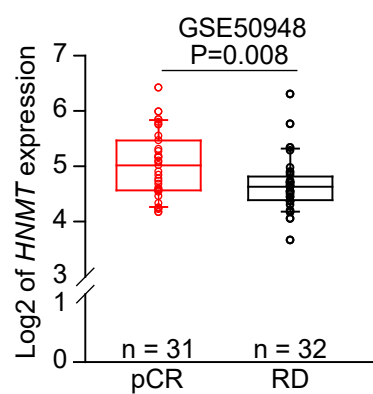

**B**

HER2+ BC (responder)

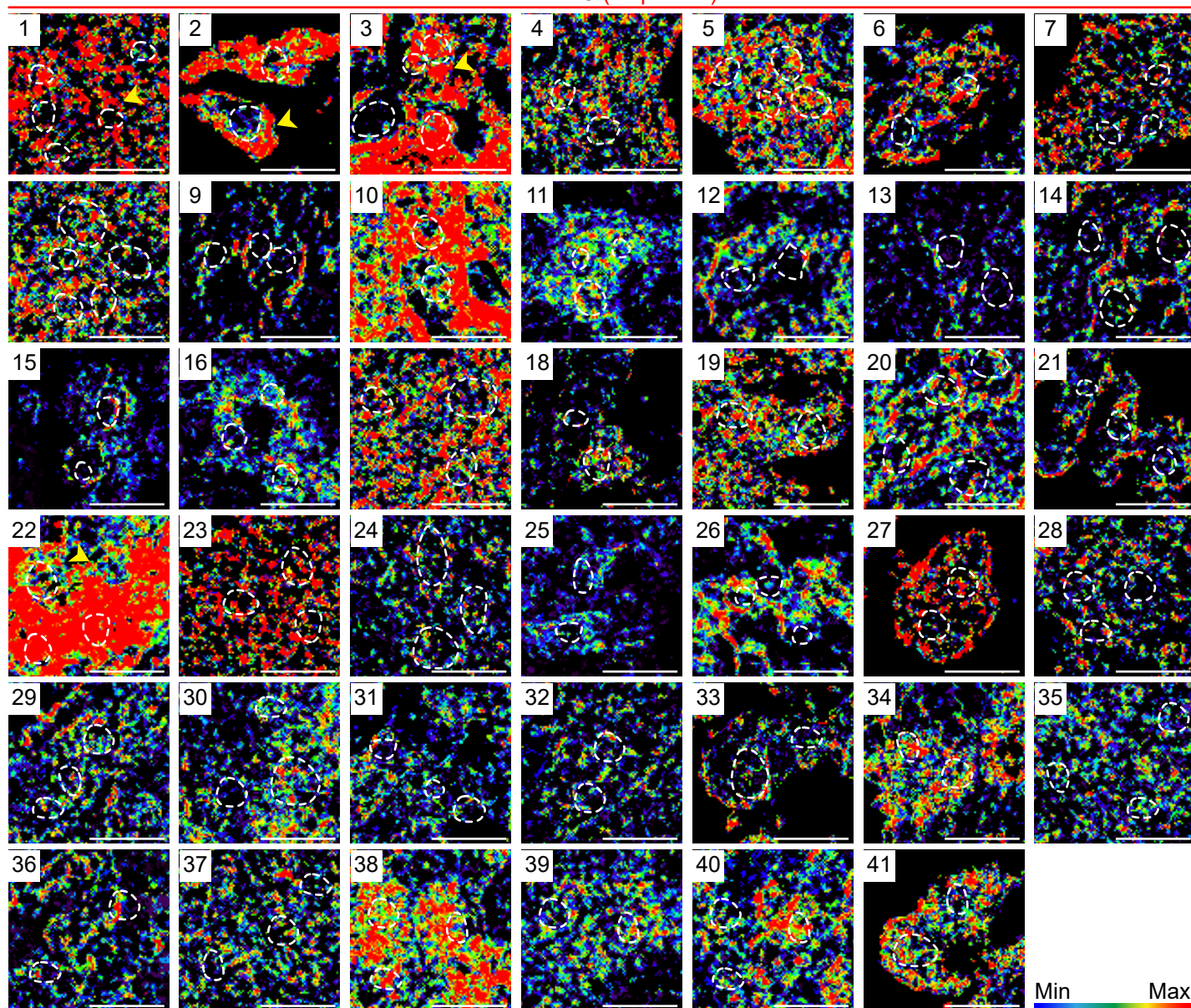

**C**

HER2+ BC (nonresponder)

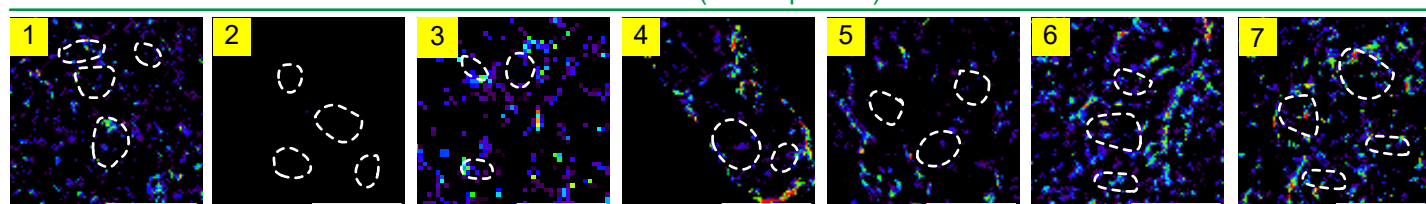

Supplement: Supplementary file 6 — Additional file 6: Fig. S6 FRET spectrum and trastuzumab responders versus non-responders in HER+ BC patients. (A) In the GSE50948 microarray dataset, HNMT protein expression was compared between the pathological complete response (pCR) and residual disease (RD) groups. The results revealed distinct expression levels, with the pCR group showing a notable increase in HNMT protein expression (depicted in red) compared to the RD group (depicted in black). The data are presented as the means ± SE. Statistical analysis was performed using the two-tailed Mann‒Whitney U test. (B-C) FRET images of HNMT and HER2-ICD protein interactions in the tumor tissues of BC patients who were trastuzumab therapy responders (n = 41) (B) and non-responders (n = 7) (C). Scale bar = 10 µm. [file 40364_2024_715_MOESM6_ESM.pdf]

**Fig. S7****A**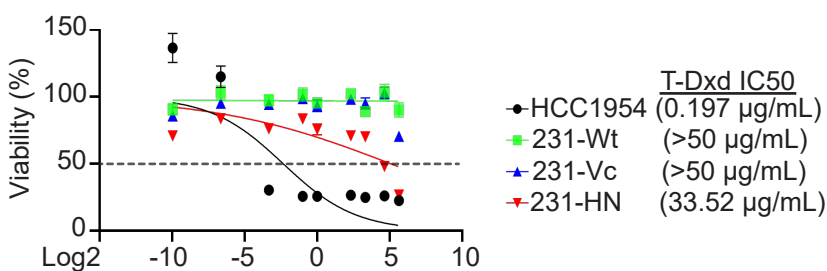**B**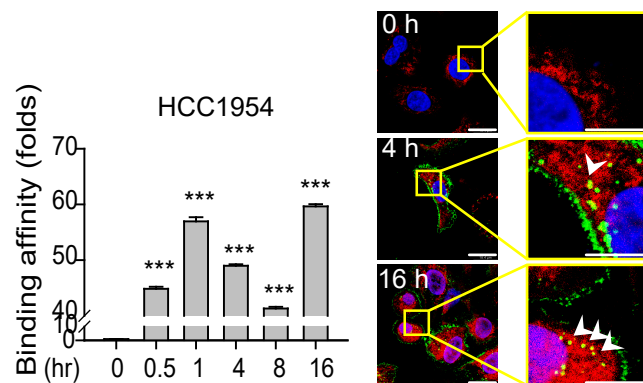**C**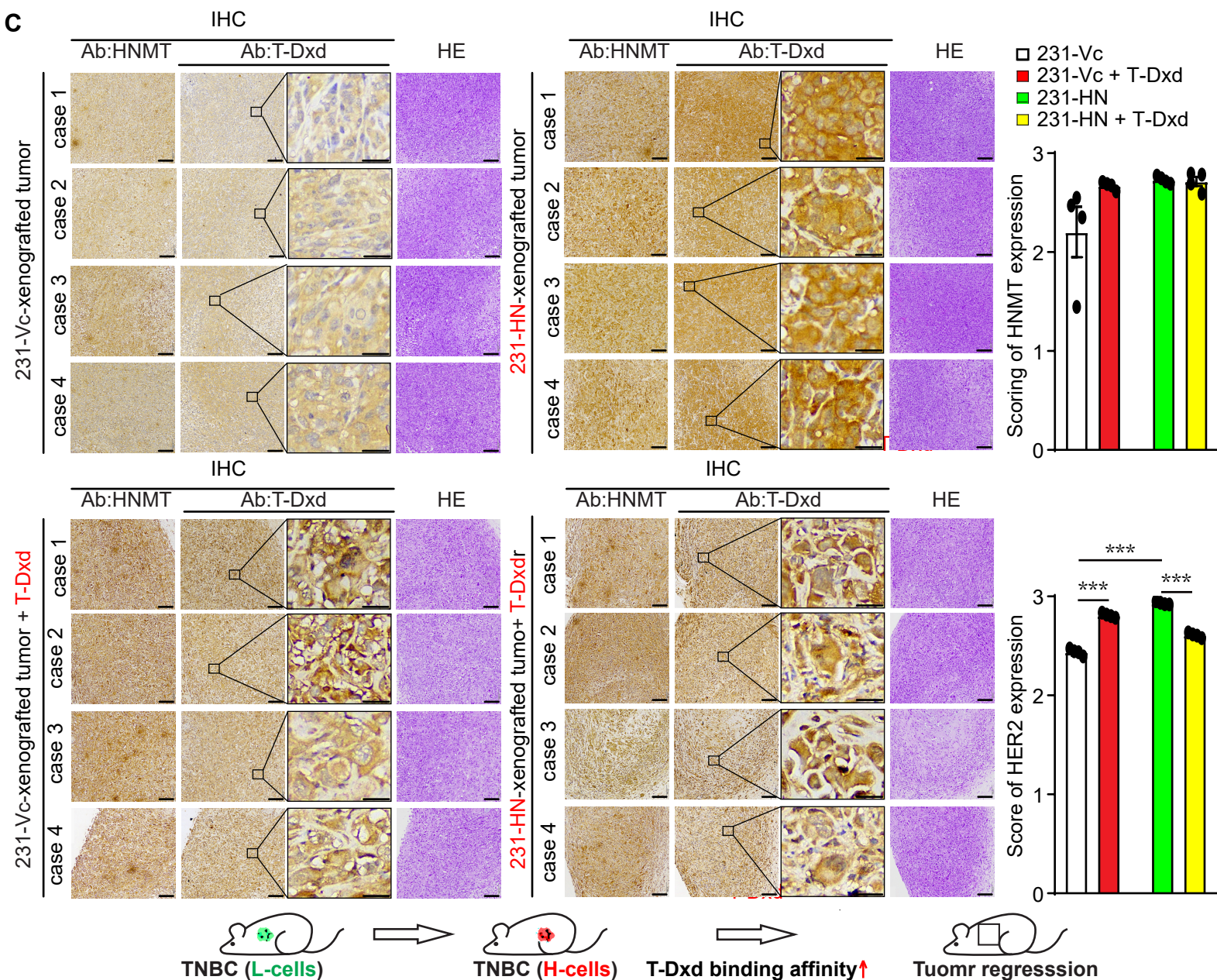**D**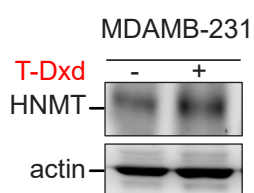**E**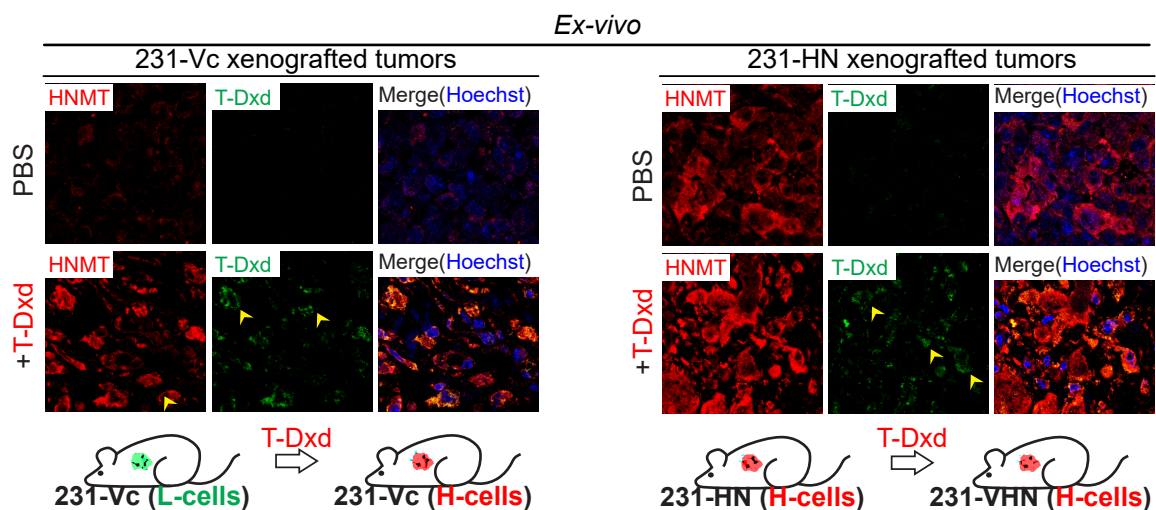

Supplement: Supplementary file 7 — Additional file 7: Fig. S7 The upregulation of HNMT expression, also referred to as the H-cell phenotype, enhances the in vivo binding affinity of T-Dxd. (A) The IC50 values of T-Dxd treatment (0, 0.01, 0.1, 0.5, 1, 5, 10, 25, and 50 μg/mL) were detected in HER2+ (HCC1954) and TNBC (231-wt, 231-Vc, and 231-HN) cell lines. (B) The time-dependent (0, 0.5, 1, 4, 8, 16 h) binding affinity of T-Dxd (10 μg/mL) was observed by flow cytometry (top) and IF staining (bottom) in HCC1954 cells. The yellow box is an enlarged image. The white arrows indicate T-Dxd located in the lysosome. The data are presented as the means ± SE. Statistical analysis was performed using a two-tailed unpaired Student's t-test. ***P < 0.001. (C) H&E and IHC were performed on TNBC (231-Vc and 231-HN) xenograft tumor tissues. The tumors were treated with/without T-Dxd (4 mg/kg). n =4 biologically independent experiments. A schematic diagram showing that T-Dxd-induced HNMT expression (H-cell phenotype) in vivo enhances T-Dxd binding affinity, resulting in tumor regression. (D) Western blotting confirmed the T-Dxd-induced expression of HNMT (H-cell phenotype) in MDA-MB-231 cells. Actin protein was used as a loading control. (E) Tumor tissue obtained from Fig. 2K was used for the ex vivo T-Dxd binding affinity assay. Representative fluorescence images of T-Dxd-treated ex vivo tumors. Yellow arrows indicate T-Dxd binding to 231-Vc- and 231-HN-derived xenograft tumors. This schematic shows that T-Dxd-induced HNMT (the L-cell phenotype transforms to the H-cell phenotype) in 231-Vc-derived xenograft tumors (in vivo) enhances the binding affinity of T-Dxd. Scale bars = 18.4 μm (B, C) and 100 μm (E). [file 40364_2024_715_MOESM7_ESM.pdf]

**Fig. S8****A**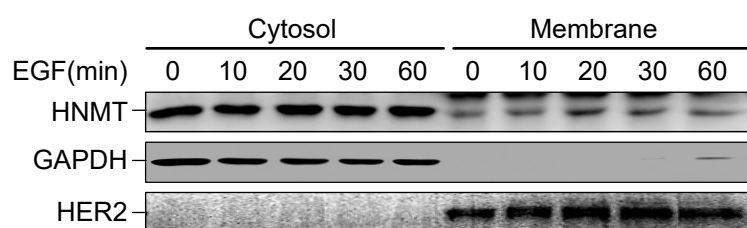**B**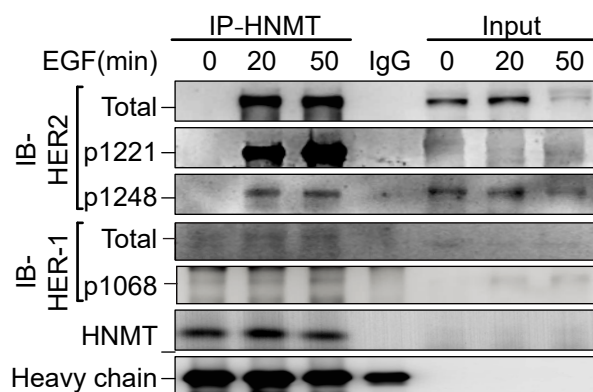**C**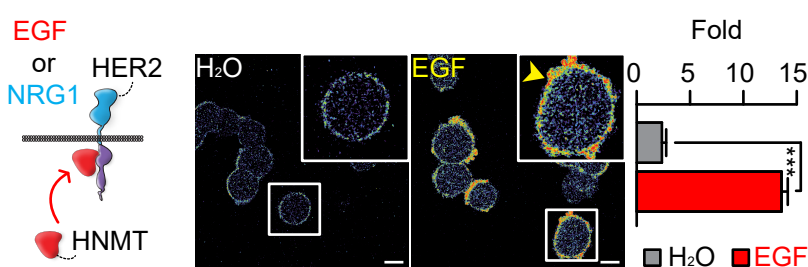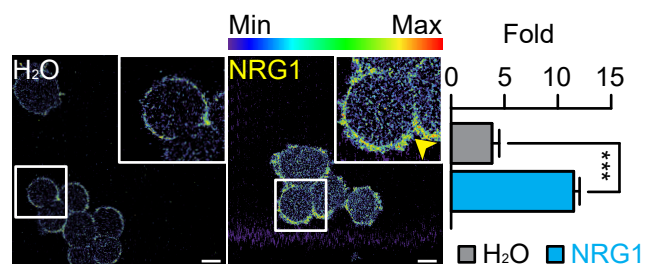**D**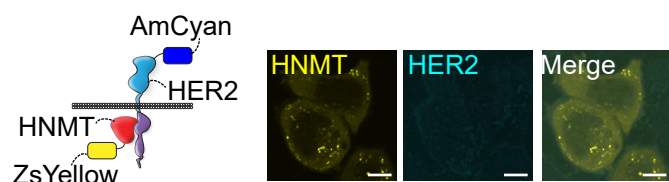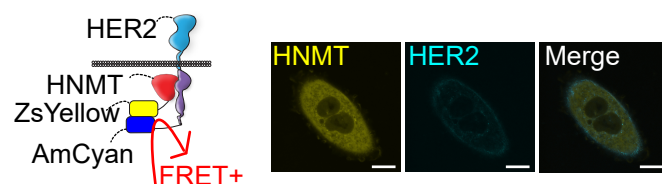**E**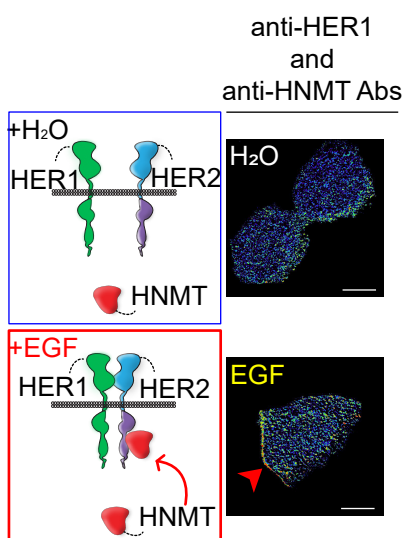**F**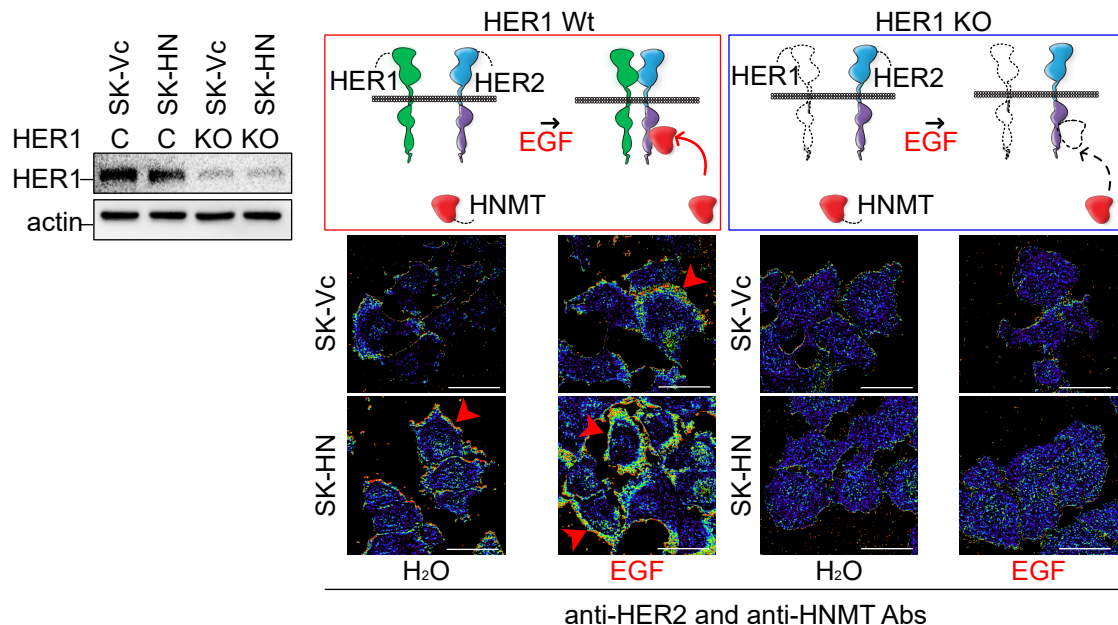

Supplement: Supplementary file 8 — Additional file 8: Fig. S8 Receptor-mediated interactions between HER2 and HNMT in BC cells. (A) BC cells were treated with EGF (100 ng/mL) in a time-dependent manner (0, 10, 20,30, and 60 min), and immunoblot analysis revealed HNMT protein expression in subcellular fractions. (B) Proteins isolated from EGF-treated SKBR3 cells in a time-dependent manner (0, 20, and 50 min), and the cells were immunoprecipitated to observe the interaction between the HNMT, HER1 and HER2 proteins. (C) Schematic representation of the ligand-mediated interaction between the HNMT and HER2 proteins at the cell membrane (left) at 20 min. Representative images and quantitative results of FRET efficiency in SKBR3 cells treated with and without EGF or NRG (100 ng/mL and 100 μM, respectively). Yellow arrows indicate a positive FRET signal. The data are presented as the means ± SE. Statistical analysis was performed using a two-tailed unpaired Student's t-test. ***P < 0.001. (D) The Supplementary Data of Fig. 3A shows schematic representations of molecular interactions and IF images with positive (right) or negative (left) FRET signals. (E) After treating SKBR3 cells with EGF (100 ng/mL) for 20 min, the FRET technique was employed to analyze the potential interaction between HER1 and HNMT. (F) SKBR3 cells were transiently transfected with CRISPR control and HER1 KO plasmids. Western blot analysis confirmed the expression of the indicated proteins in SKBR3 stable cells (SK-Vc and SK-HN). After the cells were treated with and without EGF (100 ng/mL) for 20 min, the interaction between HNMT and HER2 protein on the cell membrane was observed. It shows that the interaction between HNMT/HER2 is obvious in the presence of EGF (red arrow). Without HER1 protein, the interaction between HNMT and HER2 protein is blocked. Scale bar=10 µm (C, D, E, F). [file 40364_2024_715_MOESM8_ESM.pdf]

Fig. S10

A

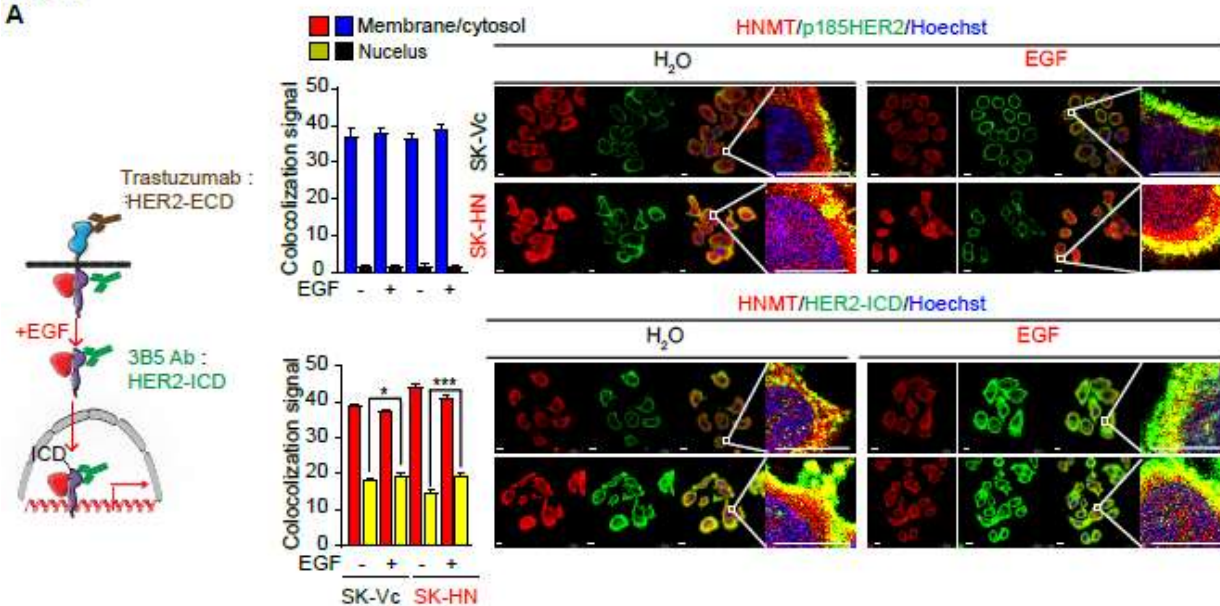

B

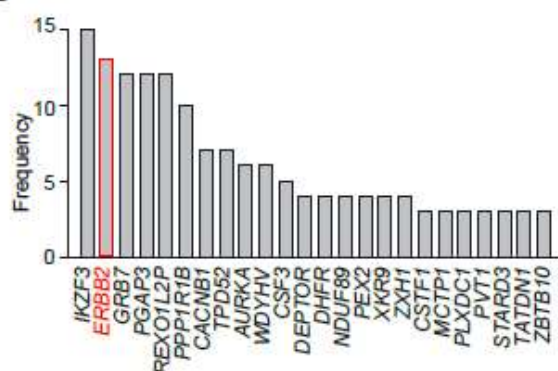

C

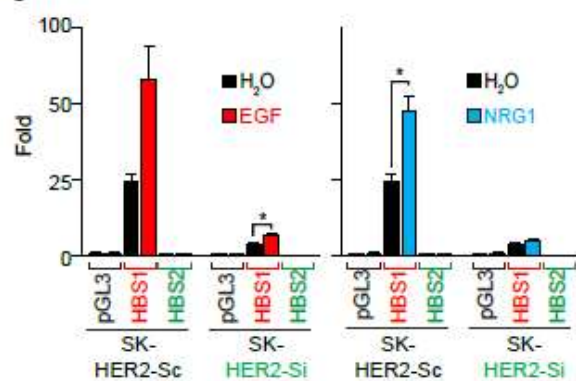

D

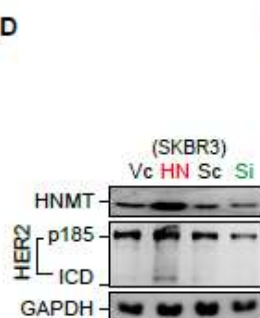

E

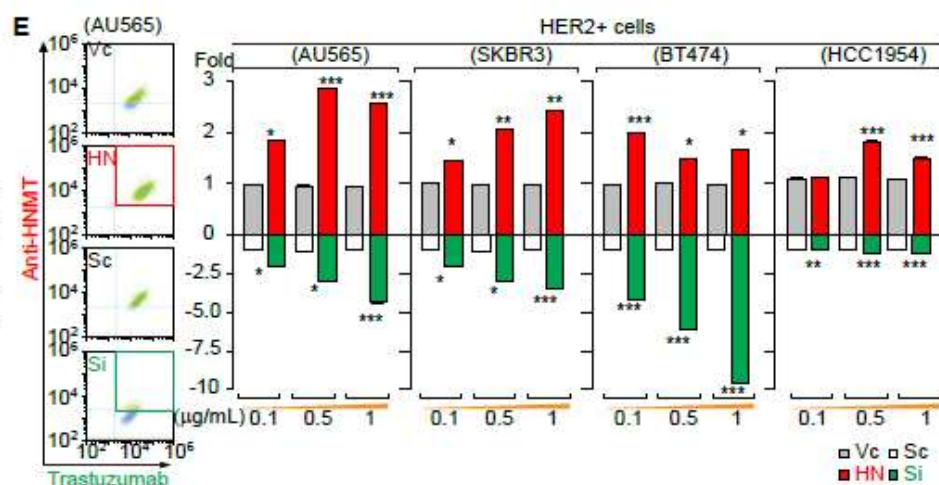

Supplement: Supplementary file 10 — Additional file 10: Fig. S10 HNMT expression affects the binding of HER2+ tumor cells to trastuzumab. (A) The cellular localization of different parts of the HER2 protein was confirmed by IF staining. Schematic representation of regions recognized by trastuzumab and anti-HER2-ICD antibodies (left). Representative images and qanitification data of IF double-stained cells showing the cellular distribution of HNMT and HER2 isoforms by 100 ng/mL EGF treating 20 min. The right panel shows HER2-ICD detection using the HER2-ICD antibody (green) and p185HER2 detection using trastuzumab (green). The white arrows indicate the nuclear localization of HER2-ICD, while the yellow arrows indicate negative results. n=5 biologically independent experiments. Scale bar = 10 µm. (B) The frequencies of the top 50 ranked target genes were determined by ChIP-sequencing analysis. (C) pGL3-4X-HBS-1 and pGL3-4X-HBS-2 were transfected into SKBR3 cells expressing the HER2 scramble or HER2 siRNA, respectively. Quantitative luc reporter assays were performed following the treatment of transfected cells with/without EGF or NRG1. The pGL3 vector was transfected into SKBR3 cells as a control. The pRL-TK plasmid was used as an internal control. (D) Western blotting confirmed HNMT and HER2 protein expression in the HER2+ BC cell lines harboring vectors overexpressing HNMT, HNMT scrambled RNA or HNMT siRNA. (E) The ability of trastuzumab to bind to the HER2 protein in the above four cell lines (D) was detected by flow cytometry. n = 3 biologically independent experiments. The data are presented as the mean ± SE. Statistical analysis was performed using a two-tailed unpaired Student's t-test. *P < 0.05, **P < 0.01, and ***P < 0.001. [file 40364_2024_715_MOESM10_ESM.pdf]

Fig. S11

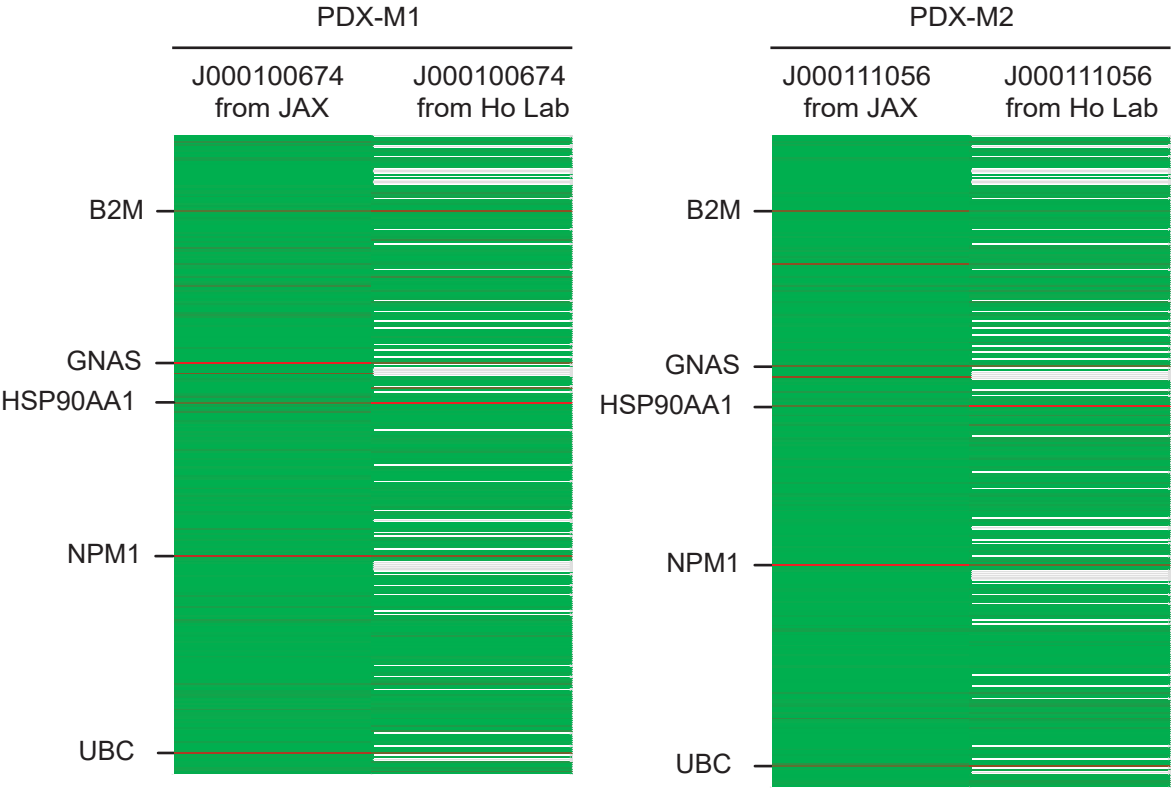

Supplement: Supplementary file 11 — Additional file 11: Fig. S11 Genetic background verification of PDX tumor tissue based on RNA sequencing data. Reference heatmaps of RNA sequencing data from both JAX and our laboratory are available for comparison. The colored lines represent individual genes. Consistent gene expression is depicted in the heatmap. [file 40364_2024_715_MOESM11_ESM.pdf]
