# Supplementary material for: Histamine N-methyltransferase (HNMT) as a potential auxiliary biomarker for predicting adaptability to anti-HER2 drug treatment in breast cancer patients
Source: Biomark Res. 2025 Jan 9;13:7. doi: 10.1186/s40364-024-00715-5 (PMC11720525; doi:10.1186/s40364-024-00715-5)
Supplement: Supplementary file 9 — Additional file 9: Fig. S9 HNMT is involved in γ-secretase-induced fragmentation of the HER2 protein, leading to cytosolic HER2-ICD shedding. (A) Schematic diagram of the γ-secretase-mediated enzymatic cleavage site in the HER2 protein sequence [41]. (B) Schematic illustration of HER2-ICD cleavage by HNMT-activated γ-secretase (left). Western blotting confirmed the expression of the indicated proteins in CHX-treated (20 μg/mL) SK-Vc, SK-HN, SK-Sc, and SK-Si cells (right) in a time-dependent manner (0, 24, and 48 h). (C) SK-Vc, SK-HN, SK-Sc, and SK-Si cell lysates were immunoprecipitated for HNMT and immunoblotted for relevant proteins as indicated. (D)Western blotting confirmed protein expression in SKBR3 cells containing scrambled PS1/2 and PS1/2 siRNA treated with or without EGF (100 ng/mL) for 24 h. (E) The expression of the indicated proteins in SKBR3 cells treated with or without CHX (20 μg/mL) at 24 h and containing scrambled PS1 and PS2 sequences, PS1 siRNA, or PS2 siRNA was confirmed by Western blotting. [file 40364_2024_715_MOESM9_ESM.pdf]

Fig. S9

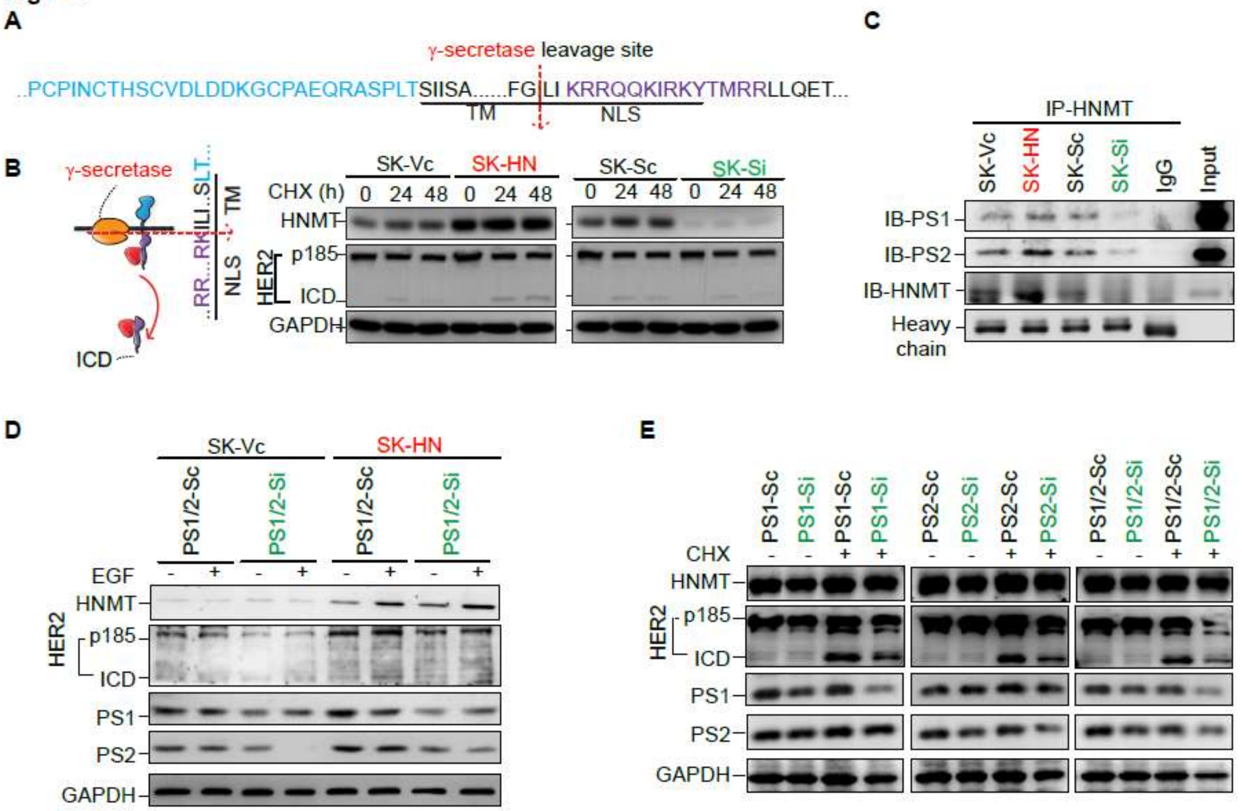
A
γ-secretase leavage site
..PCPINCTHSCVDLDDKGCPAEQRASPLTSIISA......FG LI KRRQQKIRKYTMRRLLQET...
TM
NLS
B
γ-secretase
ICD
..SLT...
LI
RK
..RR...
TM
NLS
SK-Vc
SK-HN
SK-Sc
SK-Si
CHX (h)
0 24 48 0 24 48 0 24 48 0 24 48
HNMT
HER2
p185
ICD
GAPDH
C
IP-HNMT
SK-Vc
SK-HN
SK-Sc
SK-Si
IgG
Input
IB-PS1
IB-PS2
IB-HNMT
Heavy chain
D
SK-Vc
SK-HN
PS1/2-Sc
PS1/2-Si
PS1/2-Sc
PS1/2-Si
EGF
- + - + - + - +
HNMT
HER2
p185
ICD
PS1
PS2
GAPDH
E
PS1-Sc
PS1-Si
PS1-Sc
PS1-Si
PS2-Sc
PS2-Si
PS2-Sc
PS2-Si
PS1/2-Sc
PS1/2-Si
PS1/2-Sc
PS1/2-Si
CHX
- - + + - - + + - - + +
HNMT
HER2
p185
ICD
PS1
PS2
GAPDH
