# Supplementary material for: Histamine N-methyltransferase (HNMT) as a potential auxiliary biomarker for predicting adaptability to anti-HER2 drug treatment in breast cancer patients
Source: Biomark Res. 2025 Jan 9;13:7. doi: 10.1186/s40364-024-00715-5 (PMC11720525; doi:10.1186/s40364-024-00715-5)
Supplement: Supplementary file 5 — Additional file 5: Fig. S5 H- and L-cell phenotypes visualized by IHC staining in BC tumor tissue. (A-C) H&E , HER2 IHC, and HNMT/HER2 dual IHC staining of HER2+ BC tumor tissues (A), PDX tumors (B), and HNMT-overexpressing TNBC cell xenograft tumors (MDA-MB-231 (top) and HCC38 (bottom)) (C). Scale bar = 20 µm. [file 40364_2024_715_MOESM5_ESM.pdf]

Fig. S5

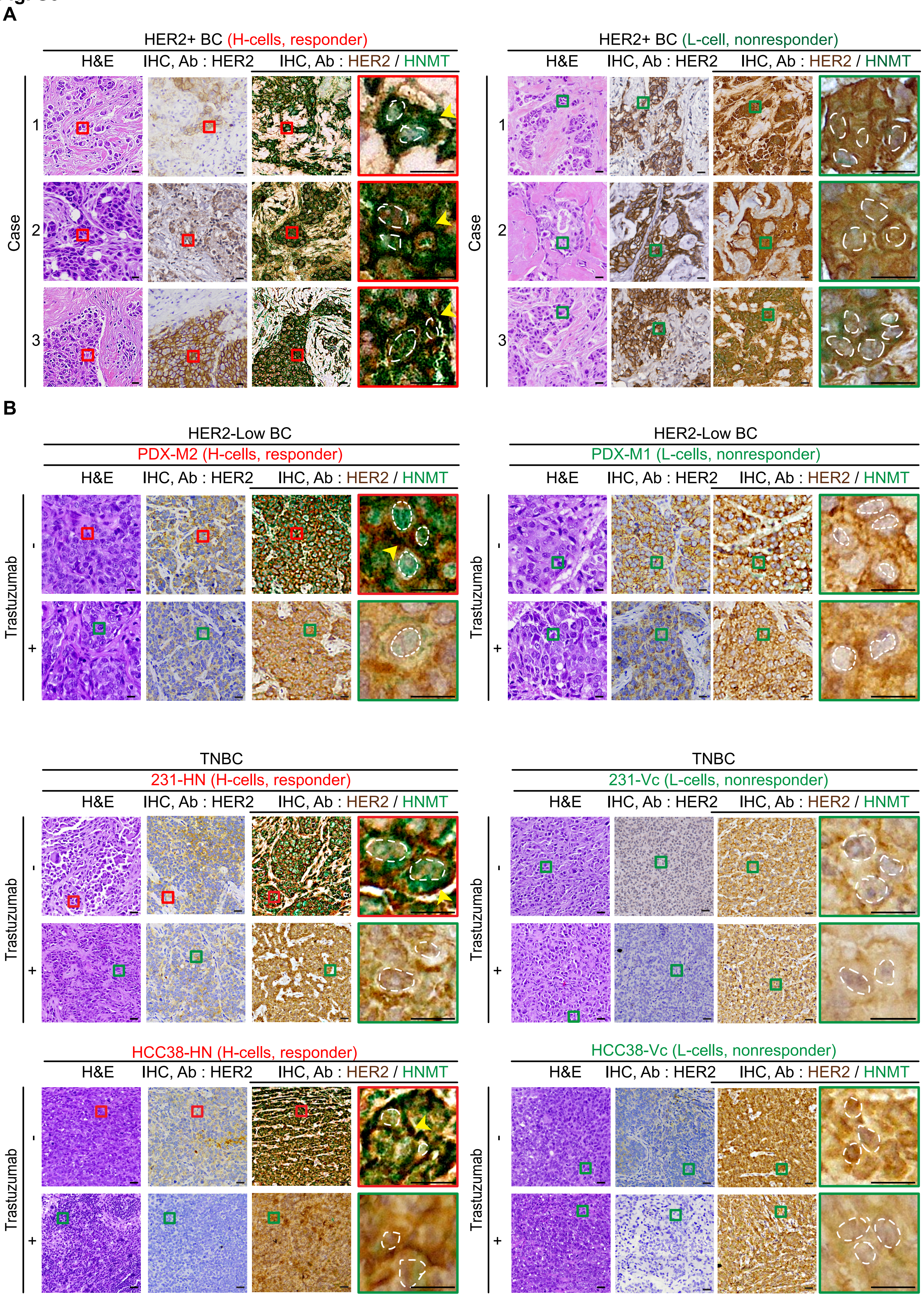
A
HER2+ BC (H-cells, responder)
H&E
IHC, Ab : HER2
IHC, Ab : HER2 / HNMT
Case
1
2
3
HER2+ BC (L-cell, nonresponder)
H&E
IHC, Ab : HER2
IHC, Ab : HER2 / HNMT
Case
1
2
3
B
HER2-Low BC
PDX-M2 (H-cells, responder)
H&E
IHC, Ab : HER2
IHC, Ab : HER2 / HNMT
Trastuzumab
-
+
HER2-Low BC
PDX-M1 (L-cells, nonresponder)
H&E
IHC, Ab : HER2
IHC, Ab : HER2 / HNMT
Trastuzumab
-
+
TNBC
231-HN (H-cells, responder)
H&E
IHC, Ab : HER2
IHC, Ab : HER2 / HNMT
Trastuzumab
-
+
TNBC
231-Vc (L-cells, nonresponder)
H&E
IHC, Ab : HER2
IHC, Ab : HER2 / HNMT
Trastuzumab
-
+
HCC38-HN (H-cells, responder)
H&E
IHC, Ab : HER2
IHC, Ab : HER2 / HNMT
Trastuzumab
-
+
HCC38-Vc (L-cells, nonresponder)
H&E
IHC, Ab : HER2
IHC, Ab : HER2 / HNMT
Trastuzumab
-
+
